# Supplementary material for: It depends on how you tell: a qualitative diagnostic analysis of the implementation climate for community-wide mass drug administration for soil-transmitted helminth
Source: BMJ Open. 2022 Jun 14;12(6):e061682. doi: 10.1136/bmjopen-2022-061682 (PMC9198697; doi:10.1136/bmjopen-2022-061682)
Supplement: Supplementary data [file bmjopen-2022-061682supp001.pdf]

Appendix 1. Qualitative interview guides

Interview guide #1: Health Centre & DeWorm3 Supervisory Teams

	Question
**1**	What kind of information or evidence are you aware of that shows whether or not community-wide MDA for interrupting transmission of STH will work in your setting?<br><br>*Follow-up questions:*<br>• *Is this evidence strong or weak?*<br>• *What evidence are you aware of from your own research? Practice guidelines? Published literature? Other settings?*<br>• *How does this knowledge affect your perception of the intervention?*
**2**	What advantages does community-wide MDA for STH have compared to school-age targeted programs? What disadvantages?
**3**	When delivering community-wide MDA for STH, what local adaptations should be made so that the intervention is effective?<br><br>*Follow-up questions:*<br>• *What aspects of the intervention should not be adapted?*<br>• *Do you think you will be able to make these adaptations? Why or why not?*
**4**	How complicated is delivery of the entire community-wide MDA for STH intervention? Please consider the following aspects of the intervention: duration of an MDA round, target population, and number of steps and sub-activities involved to fully implement.
**5**	What supportive materials or tools are needed to ensure effective implementation of community-wide MDA for STH?
**6**	What community-wide MDA for STH activities are built upon the previous LF MDA program infrastructure and experiences?<br><br>*Follow-up questions:*<br>• If none, what LF program activities could be leveraged for community-wide MDA for STH?
**7**	From your perspective, what are unique costs of implementing community-wide MDA for STH, relative to the standard of care of school-age targeted MDA?
**8**	What barriers might community members face in participating in community-wide MDA for STH?<br><br>*Follow-up question:*<br>• *What could be done to overcome these barriers?*
**9**	What kinds of infrastructure changes to the health system will be needed to accommodate community-wide MDA for STH?<br><br>*Follow-up questions:*

	Question
	• *If community-wide MDA for STH is scaled-up after the DeWorm3 project, will there need to be any changes in formal policies? Changes in information systems or data reporting systems? Other?*<br>• *What kind of approvals do you think will be needed to transition the STH program entirely to community-wide MDA? Who will need to be involved?*<br>• *Can you describe the process that will be needed to make these changes?*
**10**	How accepting are your co-workers of implementing community-wide MDA for STH? Why?
**11**	Do you think community-wide MDA for STH could be integrated into routine MOH programs?<br><br>*Follow-up questions:*<br>• If yes, how? If no, why not?<br>• *What would the effects of integration on routine programs be?*
**12**	How important do you think it is to implement community-wide MDA for STH compared to the other health priorities?
**13**	Do you feel incentivized to ensure that the DeWorm3 trial is successful in interrupting transmission of STH? What is the incentive?
**14**	How supportive are government and non-governmental leadership of implementing community-wide MDA for STH?<br><br>*Follow-up question:*<br>• *How do you think it will affect uptake/implementation?*
**15**	What kind of relevant training will you have during this intervention? Do you feel the training will prepare you to carry out the roles and responsibilities expected of you? How so?<br><br>*Follow-up questions:*<br>• *What are the positive aspects of the training?*<br>• *What is missing?*<br>• *What kind of continued training is necessary?*
**16**	Do you think that community-wide MDA could interrupt STH transmission (i.e. stop the spread of intestinal worms) in your setting? Why or why not?
**17**	How confident are you that you will be able to successfully carry out your DeWorm3 related duties? What gives you that level of confidence (or lack of confidence)?
**18**	Can you describe your team's plan for implementing community-wide MDA for STH?<br><br>*Follow-up questions:*<br>• *Do you think everyone involved understands the plan well, or is it too complicated?*<br>• *What do you do if you have to modify or revise the plan due to challenges, errors, or mistakes?*
**19**	Who are the key influential individuals or organizations to get buy-in from during the DeWorm3 study?

	Question
**20**	How should community members be informed about community-wide MDA or other DeWorm3 interventions before they occur?
**21**	What are some strategies to achieve high MDA treatment coverage?
**22**	Is there any other information that you would like to share about community-wide MDA today?

Interview Guide #2: Community Drug Distributors & Community Health Workers

#	Question
**1**	What advantages does community-wide MDA for STH have compared to school-age targeted programs? What disadvantages?
**2**	When delivering community-wide MDA for STH, what local adaptations should be made so that the intervention is effective?<br>*Follow-up questions:*<br>• What aspects of the intervention should not be adapted?<br>• Do you think you will be able to make these adaptations? Why or why not?
**3**	How complicated is delivery of the entire community-wide MDA for STH intervention? Please consider the following aspects of the intervention: duration of an MDA round, target population, and number of steps and sub-activities involved to fully implement.
**4**	What supportive materials or tools are needed to ensure effective implementation of community-wide MDA for STH?
**5**	From your perspective, what are unique costs of implementing community-wide MDA for STH, relative to the standard of care of school-age targeted MDA?
**6**	What barriers might community members face in participating in community-wide MDA for STH?<br>*Follow-up question:*<br>• *What could be done to overcome these barriers?*
**10**	How accepting are your co-workers of implementing community-wide MDA for STH? Why?
**11**	Do you think community-wide MDA for STH could be integrated into routine MOH programs?<br>*Follow-up questions:*<br>• *If yes, how? If no, why not?*<br>• *What would the effects of integration on routine programs be?*
**12**	How important do you think it is to implement community-wide MDA for STH compared to the other health priorities?
**13**	Do you feel incentivized to ensure that the DeWorm3 trial is successful in interrupting transmission of STH? What is the incentive?
**14**	How supportive are government and non-governmental leadership of implementing community-wide MDA for STH?

#	Question
	Follow-up question:<br>• *How do you think it will affect uptake/implementation?*
**15**	What kind of relevant training will you have during this intervention? Do you feel the training will prepare you to carry out the roles and responsibilities expected of you? How so?<br>*Follow-up questions:*<br>• *What are the positive aspects of the training?*<br>• *What is missing?*<br>• *What kind of continued training is necessary?*
**16**	Do you think that community-wide MDA could interrupt STH transmission (i.e. stop the spread of intestinal worms) in your setting? Why or why not?
**17**	How confident are you that you will be able to successfully carry out your DeWorm3 related duties? What gives you that level of confidence (or lack of confidence)?
**18**	Can you describe your team's plan for implementing community-wide MDA for STH?<br>*Follow-up questions:*<br>• *Do you think everyone involved understands the plan well, or is it too complicated?*<br>• *What do you do if you have to modify or revise the plan due to challenges, errors, or mistakes?*
**19**	Who are the key influential individuals or organizations to get buy-in from during the DeWorm3 study?
**20**	How should community members be informed about community-wide MDA or other DeWorm3 interventions before they occur?
**21**	What are some strategies to achieve high MDA treatment coverage?
**22**	Is there any other information that you would like to share about community-wide MDA today?

Interview Guide #3: Community members

#	Question
**1**	What advantages does community-wide MDA for STH have compared to school-age targeted programs?<br>*Follow-up question:*<br>• *What disadvantages?*
**2**	When delivering community-wide MDA for STH, what local adaptations should be made so that the intervention is effective?<br>*Follow-up question:*<br>• *What aspects of the intervention should not be adapted?*

#	Question
**3**	What barriers might community members face in participating in community-wide MDA for STH?<br><br>*Follow-up question:*<br>• *What could be done to overcome these barriers?*
**4**	Do you remember the lymphatic filariasis (LF) MDA programs that used to occur in this area? What do you remember about them?<br><br>*Follow-up questions:*<br>• *What could be done to overcome these barriers?*<br>• *Did you participate in those treatment days by swallowing the medicines given to you? Why or why not?*<br>• *Has that affected your interest in participating in the current mass treatment days?*
**5**	Do you think that community-wide MDA could interrupt STH transmission (i.e. stop the spread of intestinal worms) in your setting? Why or why not?
**6**	Who are the key influential individuals or organizations to get buy-in from during the DeWorm3 study?<br><br>*Follow-up questions:*<br>• *Can you provide specific examples of why you think their buy-in is important?*
**7**	How should community members be informed about community-wide MDA or other DeWorm3 interventions before they occur?
**8**	What are some strategies to achieve high MDA treatment coverage?
**9**	Is there any other information that you would like to share about community-wide MDA today?
