# Supplementary material for: It depends on how you tell: a qualitative diagnostic analysis of the implementation climate for community-wide mass drug administration for soil-transmitted helminth
Source: BMJ Open. 2022 Jun 14;12(6):e061682. doi: 10.1136/bmjopen-2022-061682 (PMC9198697; doi:10.1136/bmjopen-2022-061682)
Supplement: Author's manuscript [file bmjopen-2022-061682.draft_revisions.pdf]

BMJ Open is committed to open peer review. As part of this commitment we make the peer review history of every article we publish publicly available.

When an article is published we post the peer reviewers' comments and the authors' responses online. We also post the versions of the paper that were used during peer review. These are the versions that the peer review comments apply to.

The versions of the paper that follow are the versions that were submitted during the peer review process. They are not the versions of record or the final published versions. They should not be cited or distributed as the published version of this manuscript.

BMJ Open is an open access journal and the full, final, typeset and author-corrected version of record of the manuscript is available on our site with no access controls, subscription charges or pay-per-view fees (http://bmjopen.bmj.com).

If you have any questions on BMJ Open's open peer review process please email info.bmjopen@bmj.com

BMJ Open

"It depends on how you tell": A diagnostic analysis of the implementation climate for community-wide mass drug administration for soil-transmitted helminths

Journal:	*BMJ Open*
Manuscript ID	bmjopen-2022-061682
Article Type:	Original research
Date Submitted by the Author:	11-Feb-2022
Complete List of Authors:	Avokpaho, Euripide; Institut de Recherche Clinique du Benin<br>Lawrence, Sarah; University of Washington, Pediatrics<br>Roll, Amy; University of Washington, Department of Global Health<br>Titus, Angelin; Christian Medical College Vellore<br>Jacob, Yesudoss; Christian Medical College Vellore<br>PUTHUPALAYAM KALIAPPAN, SARAVANAKUMAR; Christian Medical College Vellore, Division of Gastrointestinal Sciences<br>Gwayi-Chore, Marie Claire; University of Washington, Department of Global Health; The DeWorm3 Project<br>Chabi, Félicien; Institut de Recherche Clinique du Bénin<br>Togbevi, Comlanvi; Institut de Recherche Clinique du Bénin<br>Elijan, Abiguel Belou; Institut de Recherche Clinique du Bénin<br>Nindi, Providence; Blantyre Institute for Community Outreach<br>Walson, Judd L; University of Washington, Department of Global Health; The DeWorm3 Project<br>Ajjampur, Sitara; Christian Medical College Vellore, The Wellcome Trust Resaearch Laboratory, Division of Gastrointestinal Sciences<br>Ibikounle, Moudachirou; Institut de Recherche Clinique du Benin; Universite d'Abomey-Calavi, Centre de Recherche pour la lutte contre les Maladies Infectieuses Tropicales (CReMIT/TIDRC)<br>Kalua, Khumbo; Blantyre Institute for Community Outreach<br>Aruldas, Kumudha; Christian Medical College Vellore<br>Means, Arianna; University of Washington, Department of Global Health; The DeWorm3 Project
Keywords:	Public health < INFECTIOUS DISEASES, Tropical medicine < INFECTIOUS DISEASES, Community child health < PAEDIATRICS, QUALITATIVE RESEARCH

SCHOLARONE™
Manuscripts

BMJ

I, the Submitting Author has the right to grant and does grant on behalf of all authors of the Work (as defined in the below author licence), an exclusive licence and/or a non-exclusive licence for contributions from authors who are: i) UK Crown employees; ii) where BMJ has agreed a CC-BY licence shall apply, and/or iii) in accordance with the terms applicable for US Federal Government officers or employees acting as part of their official duties; on a worldwide, perpetual, irrevocable, royalty-free basis to BMJ Publishing Group Ltd ("BMJ") its licensees and where the relevant Journal is co-owned by BMJ to the co-owners of the Journal, to publish the Work in this journal and any other BMJ products and to exploit all rights, as set out in our licence.

The Submitting Author accepts and understands that any supply made under these terms is made by BMJ to the Submitting Author unless you are acting as an employee on behalf of your employer or a postgraduate student of an affiliated institution which is paying any applicable article publishing charge ("APC") for Open Access articles. Where the Submitting Author wishes to make the Work available on an Open Access basis (and intends to pay the relevant APC), the terms of reuse of such Open Access shall be governed by a Creative Commons licence – details of these licences and which Creative Commons licence will apply to this Work are set out in our licence referred to above.

Other than as permitted in any relevant BMJ Author's Self Archiving Policies, I confirm this Work has not been accepted for publication elsewhere, is not being considered for publication elsewhere and does not duplicate material already published. I confirm all authors consent to publication of this Work and authorise the granting of this licence.

For peer review only

***It depends on how you tell*: A diagnostic analysis of the implementation climate for community-wide mass drug administration for soil-transmitted helminths**

Euripide Avokpaho[1†], Sarah Lawrence[2†], Amy Roll[2,3], Angelin Titus[4], Yesudoss Jacob[4], Saravanakumar Puthupalayam Kaliappan[4], Marie Claire Gwayi-Chore[2,3], Félicien Chabi[1], Comlanvi Innocent Togbevi[1], Abiguel Belou Elijan[1], Providence Nindi[5], Judd L Walson[2], Sitara Swarna Rao Ajjampur[4], Moudachirou Ibikounlé[1,6], Khumbo Kalua[5], Kumudha Aruldas[4], Arianna Rubin Means[*2,3]

† shared first authorship

[1] Institut de Recherche Clinique du Bénin, Abomey-Calavi, Bénin

[2] Department of Global Health, University of Washington, Seattle, WA, USA

[3] The DeWorm3 Project, Seattle, Washington, USA

[4] The Wellcome Trust Research Laboratory, Division of Gastrointestinal Sciences, Christian Medical College, Vellore, India

[5] Blantyre Institute for Community Outreach, Blantyre, Malawi

[6] Centre de Recherche pour la lutte contre les Maladies Infectieuses Tropicales (CReMIT/TIDRC), Université d'Abomey-Calavi, Benin

* Corresponding author

Email: aerubin@uw.edu

Word count: 5493

For peer review only

I, the Submitting Author has the right to grant and does grant on behalf of all authors of the Work (as defined in the below author licence), an exclusive licence and/or a non-exclusive licence for contributions from authors who are: i) UK Crown employees; ii) where BMJ has agreed a CC-BY licence shall apply, and/or iii) in accordance with the terms applicable for US Federal Government officers or employees acting as part of their official duties; on a worldwide, perpetual, irrevocable, royalty-free basis to BMJ Publishing Group Ltd ("BMJ") its licensees and where the relevant Journal is co-owned by BMJ to the co-owners of the Journal, to publish the Work in BMJ Global Health and any other BMJ products and to exploit all rights, as set out in our licence.

The Submitting Author accepts and understands that any supply made under these terms is made by BMJ to the Submitting Author unless you are acting as an employee on behalf of your employer or a postgraduate student of an affiliated institution which is paying any applicable article publishing charge ("APC") for Open Access articles. Where the Submitting Author wishes to make the Work available on an Open Access basis (and intends to pay the relevant APC), the terms of reuse of such Open Access shall be governed by a Creative Commons licence – details of these licences and which Creative Commons licence will apply to this Work are set out in our licence referred to above.

For peer review only

ABSTRACT

Objectives: Current soil-transmitted helminth (STH) morbidity control guidelines primarily target deworming of pre-school and school-age children. Emerging evidence suggests that community-wide mass drug administration (cMDA) may interrupt STH transmission. However, the success of such programs depends upon achieving high treatment coverage and uptake. This formative analysis was conducted to evaluate the implementation climate for cMDA and to determine barriers and facilitators to launch.

Settings: Prior to the launch of a cMDA trial in Benin, India, and Malawi.

Participants: Community members (adult women and men, children, and local leaders), community drug distributors (CDDs), and health facility workers.

Design: We conducted 48 focus group discussions (FGDs) with community members, 13 FGDs with CDDs, and 5 FGDs with health facility workers in twelve randomly selected clusters across the three study countries. We utilized the Consolidated Framework for Implementation Research (CFIR) to guide the design of the interview guide and thematic analysis.

Results: Across all three sites, aspects of the implementation climate that were facilitators to cMDA launch included: high community member demand for cMDA, integration of cMDA into existing vaccination campaigns and/or health services, and engagement with familiar health workers. Barriers to launching cMDA included mistrust toward medical interventions, fear of side effects, and limited perceived need for interrupting STH transmission. We include specific recommendations from community members regarding cMDA distribution sites, personnel requirements, delivery timing and incentives, leaders to engage, and methods for mobilizing participants.

Conclusions: Prior to launching the cMDA program as an alternative to school-based MDA, cMDA was found to be generally acceptable across diverse geographic and demographic settings. Community

For peer review only

For peer review only - http://bmjopen.bmj.com/site/about/guidelines.xhtml

members, CDDs, and health workers felt that engaging communities and tailoring programs to the local context are critical for success. Potential barriers may be mitigated by identifying local concerns and addressing them via targeted community sensitization prior to implementation.

Strengths and limitations of this study

- This study highlights opportunities and challenges to launching community-wide mass drug administration (cMDA) for STH as an alternative to school-based MDA from the perspective of community members and the health delivery workforce that will be responsible for delivery of future cMDA programs.
- Findings from three diverse settings strengthens opportunities to understand similarities and differences across geographic areas.
- Some participants may have heard about DeWorm3 before participating in focus-group discussions (FGDs), which may pose threats to social desirability and response biases.
- Although a large number of FGDs were conducted across heterogeneous settings, the generalizability of study findings may not be translatable to other STH-endemic areas.

INTRODUCTION

Neglected tropical diseases (NTDs) affect some of the world's most impoverished populations and contribute to a variety of morbidities that exacerbate existing health and economic inequities (1). Infections with one NTD, soil-transmitted helminths (STH), are associated with anemia, cognitive development delay, growth stunting, pre-term birth, and other adverse outcomes (2). World Health

For peer review only

Organization (WHO) guidelines recommend control of STH morbidities via annual or bi-annual deworming of pre-school and school-age children and other high-risk groups, including pregnant women and adolescent girls (3). However, in many settings, the current STH strategy would likely need to be continued until significant economic development and universal water, sanitation and hygiene (WASH) access are broadly available to stop transmission of STH (4). Emerging evidence suggests that it may be possible to interrupt transmission of STH by expanding deworming to treat individuals of all ages via community-wide MDA (cMDA) (5, 6).

The current standard of care for STH is school-based MDA to reach pre-school and school-age children, and many school-based deworming programs have been successfully implemented for decades. Transitioning from school-based MDA to cMDA for community-level STH transmission interruption will require adapting long withstanding programs or designing new platforms for reaching much larger target populations. These transitions should be approached carefully, as they will likely affect community member and health worker attitudes towards and engagement in new programs. The success of cMDA in interrupting transmission of STH is indeed predicated upon programs attaining high treatment coverage (drug receipt) and uptake (drug ingestion) (7, 8). Many STH-endemic communities have a long history of participating in other community-based mass distribution programs, including campaigns for lymphatic filariasis (LF), onchocerciasis, trachoma, malaria bed net distribution, and/or mass immunization programs such as polio campaigns (9). Factors that influence participation in mass campaigns include recipient trust in programs and efforts to tailor programs to local conditions (10). Negative campaign experiences or perceptions can compromise the success of future programs, particularly those requiring high coverage over multiple years to reach targeted transmission endpoints (11, 12).

Formative qualitative research can be used to understand community-member and implementer perceptions of past, ongoing, or prospective community-based campaigns. Diagnostic analyses, an application of formative evaluations, are particularly helpful in illuminating processes that can facilitate

For peer review only - http://bmjopen.bmj.com/site/about/guidelines.xhtml

or impede implementation. In this study, we perform a diagnostic analysis of the implementation climate to proactively identify factors influencing the launch of cMDA for STH transmission interruption, including (1) perceptions of current deworming practice, (2) potential barriers and facilitators to transitioning from school-based MDA to cMDA delivery, and (3) perceived effectiveness and need for cMDA (13).

METHODS

This analysis was conducted at the outset of the DeWorm3 Project, a large hybrid type I community cluster randomized trial in Benin, India, and Malawi (Table 1). DeWorm3 aims to determine the feasibility of interrupting STH transmission using biannual cMDA, relative to standard-of-care school-based MDA (14-16).

For peer review only

For peer review only - http://bmjopen.bmj.com/site/about/guidelines.xhtml

Table 1. Overview of study sites

	Benin	India	Malawi
**Site**	Commune of Comè	Vellore and Thiruvannamalai Districts, Tamil Nadu	Mangochi District
**Geographic area of site (km²)**	148	477	289
**Total number of households**	24,378	36,536	27,750
**Population size**	94,969	140,932	121,819
**Standard of care**	Annual school-based MDA targeting children 5-14 years of age	Biannual school-based MDA on National Deworming Days targeting children 1-19 years of age	Annual school-based MDA and Child Health Days targeting children 1-14 years of age
**cMDA workforce**	Community drug distributors (CDDs), coordinated by the Ministry of Health	CDDs and Accredited Social Health Activists (ASHA), women working as health educators and promoters in their communities	Community health workers (Health Surveillance Assistants) who also fill the rolls of CDDs, coordinating with teachers

120 **Sampling strategy**

121 Key stakeholders shaping the implementation climate for cMDA include community members and local
122 health workers. Focus group discussions (FGDs) were conducted separately with groups of community
123 members, including adult women and men (over 15 years), community leaders, and children (ages 12-15
124 years of age), local health workers, including CDDs, and Ministry of Health (MOH) health facility workers
125 who often serve as CDD supervisors.

126 Prior to trial randomization (e.g., before designations of intervention or control clusters were made), four
127 clusters were randomly selected in each site to conduct community-level FGDs. In each cluster, one FGD
128 was conducted within each community member strata (four total), two FGDs among drug distributors,
129 and one FGD among local MOH health facility workers. The sampling strategy for identifying and recruiting
130 community members for FGDs within each cluster differed slightly by site (Table 2). In India, purposive
131 sampling was employed, in which village leaders/influencers identified potential participants. In Benin and
132 Malawi, community members were randomly selected to participate from a pool of individuals who
133 attended outreach meetings at the chiefs/headmen's residence. The first five randomly approached

For peer review only - http://bmjopen.bmj.com/site/about/guidelines.xhtml

individuals from each demographic strata who agreed to participate were invited to attend FGDs within the next week (except children, for whom parents/caregivers were approached). No more than one individual per household was selected to participate in an FGD in a given cluster. Transportation was offered to individuals who needed access to the FGD location. In Benin and India, local leaders were chosen using purposive quota sampling. Purposive quota sampling was used to invite CDDs and health workers from local health facilities in each cluster to participate in FGDs.

Table 2: Sampling strategy by stakeholder group

Stakeholder	Targeted sample size (per FGD)	Sampling strategy
Community members		
Adult women (15+ years of age)	5-10	Purposive sampling (India)<br>Random sampling (Benin[1] and Malawi)
Adult men (15+ years of age)	5-10	
Local leaders	5-10	
Children (12-15 years of age)	5-10	
Health center staff and CDD supervisors	5-10	Purposive quota sampling
CDDs	10-15	Purposive quota sampling

[1] Purposive quota sampling was used to sample local leaders in Benin and India

Data collection

This diagnostic analysis study design is informed by the Consolidated Framework for Implementation Research (CFIR), a meta-theoretical framework of 38 constructs that provides a typology of constructs for characterizing potential determinants (barriers and facilitators) to implementation from the perspective of individuals involved in implementation (17). CFIR constructs are organized according to five major domains influencing implementation and implementation effectiveness including (1) the intervention, (2) the inner setting, (3) the outer settings, (4) the individuals involved, and (5) the process for accomplishing the intervention. While the CFIR can be used at any stage of implementation, when applied pre-implementation, the CFIR can help proactively identify opportunities and challenges facing implementation and inform adaptations to implementation strategies for the local context (17, 18).

For peer review only

We drew upon the CFIR to inform the design of four semi-structured interview guides with a mix of respondent and informant style questions, tailored to each stakeholder group (one question guide was used for all adult community members). In this study, we identified *a priori* 23 CFIR constructs across all five domains that we hypothesized would influence the implementation climate for cMDA and which were appropriate for use during formative research prior to implementation (Appendix 1) (18). The question guides were piloted and adapted slightly as necessary within each site to ensure that the questions were clear, meaningful, and culturally appropriate. Site adapted question guides were thereafter translated into local languages including Yao (Malawi) and Tamil (India), and the official language (French) in Benin. FGD facilitators in Benin adapted the French question guide to local languages, including Watchi and Pédah when necessary, during FGD facilitation.

All participants provided written consent prior to the start of the FGD. The parents or caregivers of participating children similarly provided written consent and children ages 12-15 also provided written assent. Consent and assent could also be provided by a thumbprint in the presence of a witness. FGDs were conducted in private locations with both a facilitator and notetaker present and all FGDs were audio-recorded with participant permission.

Analysis

Audio files were transcribed verbatim in the local or official language. For each transcript, two one-minute random spot checks were conducted on each audio file for quality assurance. All transcripts were then translated into English. All transcriptions and their translations were reviewed by a second individual fluent in both English and the local language for quality assurance.

Coders were based in each DeWorm3 site as well as at the central level (University of Washington, Seattle). For data collected in Benin and India, two primary coders were assigned to each transcript, with a third coder designated as the "tie-breaker." When possible, at least one coder was based at the site in

For peer review only - http://bmjopen.bmj.com/site/about/guidelines.xhtml

175 which the data were collected, and the other coder was a member of the DeWorm3 central team. For
176 data collected in Malawi, a single primary coder from the central level coded the data while a secondary
177 coder at the site reviewed and validated the findings, due to coder availability. Each primary coder
178 independently read and coded each transcript primarily using a deductive approach and a CFIR-based
179 codebook. Coding teams from each country and the central level met via conference calls to iteratively
180 refine code definitions and code inclusion/exclusion criteria until a final codebook was established. After
181 a transcript was coded, the coders assigned to the transcript met via conference call for consensus
182 meetings to discuss where applied codes diverged. When necessary, a third coder weighed in where
183 consensus between primary coders was not reached.

184 The final coded transcripts were used to create case memos that were grouped by stakeholder category
185 and site. The case memos included a summary of how the code was applied for a given stakeholder group,
186 a justification for the summary provided noting code patterns and latent messages, and specific quotes
187 highlighting how the code was applied. The summaries, patterns, and themes from the coded transcripts
188 and case memos were used to guide thematic analysis, an analytical method that is useful for summarizing
189 key features of large datasets using a clearly-structured approach (19, 20).

190 **Patient and public involvement**

191 Community members and health workers living in the sampled STH endemic areas were not involved in
192 design, conduct, or reporting of this qualitative study. However, all feedback from community members
193 was used to shape a subsequent community-based intervention within a larger clinical trial.

194 **Ethical approval**

195 This study has been reviewed and approved by the Institut de Recherche Clinique du Bénin (IRCB) through
196 the National Ethics Committee for Health Research (002-2017/CNERS-MS) from the Ministry of Health in
197 Benin, The London School of Hygiene and Tropical Medicine (12013), The College of Medicine Research

For peer review only - http://bmjopen.bmj.com/site/about/guidelines.xhtml

Ethics Committee (P.04/17/2161) in Malawi, and Christian Medical College, Vellore, in India (10392). The study was also approved by The Human Subjects Division at the University of Washington (STUDY00000180).

RESULTS

In this study, 48 FGDs were conducted with community members – four FGDs (one per cluster) for each stakeholder group: adult women, adult men, children, and local leaders, totaling 16 in each site, 13 with CDDs (two each in Benin and Malawi, nine in India), and five with CDD supervisors (two in Benin and three in India).

Across FGDs and settings, key themes emerged within four CFIR domains: intervention characteristics, inner settings, characteristics of individuals, and process. Factors positively influencing the implementation climate for cMDA across sites included community member demand for community-wide (versus school-based) MDA, integration of MDA into existing vaccination campaigns and/or health services, and engagement with health workers (including trained CDDs) rather than community volunteers. Factors negatively affecting the implementation climate across sites included mistrust and resistance toward medical interventions, fear of side effects, and limited perceived need. Additional process recommendations emerged as key themes that varied slightly across sites and included suggestions regarding MDA distribution sites and distributors, treatment costs/financial incentives, engaging leaders, and engaging participants through sensitization and mobilization efforts.

For peer review only

Intervention characteristics

Relative advantage: cMDA is preferable to school-based MDA

The CFIR construct of *relative advantage* captures participant perceptions regarding the benefits of implementing one intervention compared to an alternative (17). Across community member and health

worker/CDD groups and sites, participants identified a preference for cMDA as compared to school-based deworming programs for several reasons. Participants stressed that providing STH treatment to both children and adults is the only way to prevent STH reinfection.

Across stakeholder groups, participants also highlighted that children who were not enrolled in school would be able to receive treatment through cMDA. Adult community members in Benin and Malawi were particularly concerned that school-based MDA campaigns do not always provide parents with treatment information prior to distribution and often administer the medications without parental consent or trust. Additionally, they thought uptake would be improved if parents are involved in treatment administration.

> *It's better to go through the parents to reach the kids. Parents know how to approach their children, manage them and make them understand the benefit of the thing [medicine]. The child will easily take the medicine without any effect.* (Cluster 26, Women, Benin)

Across FGDs and sites, participants were enthusiastic that cMDA could interrupt STH transmission and increase parental engagement with the intervention, particularly to enable parental consent, and allow them to encourage and confirm their child's uptake.

Design quality & packaging: Door-to-door MDA delivery by highly trained, familiar distributors is preferred

Design quality describes stakeholder recommendations for how to bundle, present, and assemble the intervention (17). Across sites and stakeholder groups, campaigns that delivered services door-to-door were considered more desirable than those that used a central distribution site (Table 3). In India, community participants reflected upon past experiences with LF MDA campaigns that were door-to-door whereas participants in Benin reflected upon experiences with door-to-door vaccination campaigns and bed net distributions at local health centers. Long waiting times, associated with lost income and productivity, were identified as primary barriers to central distribution sites. One female participant in Benin reported she would not wait around all day for someone to distribute MDA but instead would just

For peer review only - http://bmjopen.bmj.com/site/about/guidelines.xhtml

purchase the medications herself, given their low costs. Additionally, participants stressed that door-to-door campaigns improve equity by increasing the likelihood of reaching those unable to travel due to financial or physical barriers.

> *The HSAs should go door by door to give people the medicine as some people, for example old and crippled, may not be able to go and access the drugs. But if they go door by door, then everyone receives the drugs and not only those who walk.* (Cluster 21, Women, Malawi)

Community members and leaders across sites, participants preferred to receive treatment from individuals perceived to be health professionals, especially a familiar health worker or CDD, or someone working with a well-respected non-governmental organization (NGO). Participants believed that increased health worker engagement could alleviate community mistrust linked with fear of adverse events by medicalizing the distribution process and making community members feel safer, thereby increase treatment coverage. Men in Malawi stressed that health volunteers are often poorly respected and mistrusted, while clinically-trained health professionals are typically more respected. Of paramount importance for adult community members and CDDs was that distributors are known members of the community.

> *When community members see new faces during a project, they tend to be resistant, so it is better to use people from the area and not strangers. If not, this may not be successful.* (Cluster 21, Local leader, Malawi)

> *But above all, it is necessary to involve health workers, who the population trusts....Many are afraid because they do not see us, they do not see the health workers on the ground.* (Health Center Staff, Benin)

In Benin, local leaders noted that when NGOs engage in cMDA, it is important they are well-respected and have well-recognized logos that community members are familiar with and trust based on their prior

For peer review only

work. Regardless if cMDA is administered by a health professional, volunteer, or NGO, community members across sites noted that their willingness to participate in cMDA is driven by their perception that they have been fully and accurately informed about cMDA, and that they have had time to ask questions.

> *Even if it [deworming medication] is given for free, they will not eat it unless it has been explained and given. If they are told they will benefit...with awareness in the villages, they will eat it.* (Cluster 12, CDD, India)

Community members expressed that they wanted to be treated with dignity and that their participation in community-wide public health campaigns of any kind should not be taken for granted.

> *Whether they eat the tablet or not it depends on to what extent this information reaches the people. It depends on how you tell.* (Cluster 34, Men, India)

> *The messages about drugs should be given to us in good time and not just tell us like today that tomorrow we will have a drug administration activity. Many people need time to ask questions and clear their myths before they get treated. Some people tend to refuse medicine because of fear of side effects, so when you sensitize them for a long time, they tend to listen and at the end the program becomes successful. So avoid short notice messages, people are difficult. They need enough time to understand what is happening.* (Cluster 21, Men, Malawi)

In Malawi, local leaders reported that community members want to be followed up with after distribution to monitor for adverse events or continued engagement with distribution programs to foster trust in future campaigns. Without this, the leaders feared that negative rumors might proliferate, or communities might feel as though they only received treatment for research purposes, rather than for their wellbeing.

Intervention complexity: cMDA is complex, but still feasible to implement

For peer review only - http://bmjopen.bmj.com/site/about/guidelines.xhtml

The CFIR construct of *complexity* is defined as the perceived difficulty of implementing an intervention (17). Across groups, participants were concerned about the timing of cMDA, the distance to distribution sites if cMDA is centrally located as opposed to delivered door-to-door, and whether or not they would have sufficient notice about cMDA before the campaign begins. Many community members suggested optimal distribution times, which varied by site depending on common work schedules and holidays. Concerns regarding health worker/CDD knowledge and accommodation of community members' schedules were prevalent across FGDs but some participants stressed they would change their schedules to be present for distribution if informed by community leaders.

> *Even if someone has a plan to go to the field or to the market, three days before the distribution of the drug, they will cancel their plan and come and listen to what the village chief invited them to do...if everyone is not informed, it [MDA] cannot succeed*. (Cluster 10, Women, Benin)

Adults and children across sites recommended that distribution over multiple days within a community to reach the greatest number of people.

> *The period of drug administration should be long so that everyone is able to receive treatment. Some people may not be home during the time that you have set to administer the drugs and as such if done for maybe only a day, it means those people will not receive the drugs. But if it is for some more days then everyone will be treated.* (Cluster 21, Men, Malawi)

Inner setting

Implementation climate: Initial mistrust of MDA is likely, but demand and perceived need will counter this

The CFIR construct *implementation climate* captures comments related to the community member's receptivity to implementation, and the extent to which implementation is supported (17). The core

For peer review only - http://bmjopen.bmj.com/site/about/guidelines.xhtml

component of implementation climate discussed across FGDs were factors that influence community member trust in treatment campaigns. Participants across the sites anticipated high levels of initial mistrust and potential resistance toward newly launched cMDA for STH. This initial mistrust is driven by personal and anecdotal evidence of adverse side-effects such as fatigue, stomachaches, and fever after previous school-based deworming MDA campaigns.

> *Other pupils received the medical treatment before they ate a meal, hence they vomited. So those that did not receive the medical treatment were afraid of vomiting too if they took the medicine.* (Cluster 19, Children, Malawi)

> *They will eat [medication] based on the trust. They will eat [medication] based on your approach, otherwise they may take and keep it aside somewhere....* (Cluster 17, Men, India)

In Malawi, limited follow-up by transitory MDA programs and research projects was also noted as fueling mistrust of community health programs. Similarly, in one CDD FGD in India, participants identified mistrust of government programs as a potential barrier to MDA campaigns where medications are provided for free. CDDs explained that community members perceive government provided medications to be of poorer quality and therefore less effective with greater risks of side effects; therefore, those who can afford to purchase their own medications from pharmacies will often do so.

> *For people who can, they will get it [deworming medicine] from the medical shop. Whatever is given through the government they will keep it aside and they will not use it.* (Cluster 15, CDD, India)

In Benin, some participants from the men's, women's, and CDD FGDs shared concerns that drugs used in such campaigns might be given by Westerners with malintent.

For peer review only

For peer review only - http://bmjopen.bmj.com/site/about/guidelines.xhtml

> *The majority of the population does not understand. They think that the drugs are poisoned in order to reduce the African population.* (Cluster 10, Men, Benin)

Similarly, in Malawi, CDDs identified rumors and misinformation as major barriers to delivering cMDA with high coverage. Specific rumors include that stool collected for STH surveillance would be used for Satanist practices, rather than medical purposes, and that school-based deworming programs provide contraceptives to young children to reduce population growth.

While participants noted that mistrust and resistance might initially be high following a transition to cMDA for STH, there was still a strong perceived demand for deworming of all ages, and a sentiment that community sensitization could overcome these concerns.

> *...we are looking forward to this [community-based MDA] and we would like this to be a regular treatment. People are suffering from intestinal worms and only children receive the treatment. So this project [DeWorm3] will help all of us to receive treatment.* (Cluster 21, Men, Malawi)

Compatibility: Community-based MDA is highly compatible with existing health infrastructure

The CFIR construct *compatibility* is highly related to *implementation climate*, capturing the alignment between the innovation and existing values and priorities (17). Health facility workers and CDDs across sites noted cMDA should be integrated into existing community programs or, at a minimum, coordinate with ongoing community-based activities to improve treatment coverage and mitigate risks of conflict with ongoing local health programs. CDDs in India and community leaders in Benin identified community-based vitamin A and iron distribution and childhood vaccination campaigns as ideal programs to integrate with cMDA. In India, CDDs also suggested integrating cMDA with existing indoor residual spraying programs for vector control.

For peer review only

For peer review only - http://bmjopen.bmj.com/site/about/guidelines.xhtml

The voluntary workers who go house to house to spray mosquitoes, we can make use of them to give the tablet...Earlier they were going once a month or once a week, but now they go daily house to house. We can give through them. (Cluster 17, CDD, India)

Available resources: Training, storage, and hygiene infrastructure are key resources for MDA implementation

The CFIR construct *available resources* describes the financial and material resources available (and desired) for implementation, including training and education, space, time, and money (17). Health facility workers and CDDs across sites stressed that existing resources may not be sufficient for delivery of cMDA. CDDs in particular were concerned about receiving adequate training and access to resources to take home for self-review. CDDs and health workers highlighted that they wanted more than a single one-day training prior to MDA, in order to provide adequate time to practice and apply skills in a training environment. Across sites, CDDs noted the importance of training before distribution.

Other key resources identified by CDDs and health workers in Benin included medication storage in the field, community education materials, shelter during inclement weather, as well as food, water, and financial incentives for CDDs. CDDs in Benin were particularly concerned with timely payment for their work. In Malawi, local leaders noted that in the past villagers have felt burdened by volunteering for health program implementation without compensation. They also noted that villagers might expect payment for participating in MDA, given past experiences with research projects providing stipends. In India, health facility workers wanted to ensure they would have adequate staffing to assist during MDA.

Lastly, CDDs and health center personnel across sites, women in Benin, and leaders in India stressed that hygiene infrastructure needs to be improved and that investing in WASH as part of a broader STH elimination program might, as a result, increase treatment coverage of cMDA by demonstrating long-term investments in community well-being.

For peer review only - http://bmjopen.bmj.com/site/about/guidelines.xhtml

> *The rules of hygiene are very important, very, very important. Without it, we cannot right away start distributing the drugs and say that we want to completely eradicate the transmission of worms, impossible.* (CDD, Benin)

Characteristics of individuals

Knowledge & beliefs: Skepticism of the clinical rationale for MDA, fear of side effects, and limited perceived need for treatment will be barriers to cMDA

The *knowledge and beliefs* CFIR construct is defined as individuals' attitudes toward and value placed on implementation and their familiarity with related facts, truths, and principles (17). While participants strongly believed cMDA could eliminate STH transmission, some reservations about MDA rooted in knowledge and perceptions about deworming medications remain. For example, adult men and children in Benin and local leaders in Malawi raised concerns about the effects of treating people who may not be infected with STH.

> *When you get the drugs and you do not have the worms, the tablet can still damage your organs such as organs of digestion or breathing.* (Cluster 1, Children, Benin)

In India and Malawi, participants in the men's FGDs thought individuals who feel healthy might perceive themselves to be at low risk of STH and thus choose not to participate in cMDA. Women in Malawi reported this occurred during prior cMDA campaigns while women in India also noted individuals with limited literacy might not understand the need for treatment and be reluctant to participate in MDA campaigns.

Self-efficacy: Community members and CDDs will be key mobilizers for cMDA

The CFIR construct *self-efficacy* captures comments that reflect an individual's beliefs in their own abilities to achieve implementation goals (17). Women and community leaders in Benin identified themselves as

For peer review only - http://bmjopen.bmj.com/site/about/guidelines.xhtml

important catalysts in influencing the acceptability of MDA by working together and influencing their social networks.

> *The process is simple as we have just understood, we will also explain to our brothers and sisters who will not accept that treatment is a good thing. We will tell them they should not be discouraged adding that there is good in it. It's up to us to explain to them.* (Cluster 1, Women, Benin)

Health facility workers and CDDs also identified themselves as important contributors to ensuring successful MDA implementation, given their existing relationships with communities.

> *We worked with them and they know us on the ground to be able to do the job, so there are no issues for community health workers.* (CDD, Benin)

Process

Engaging: Local leaders and sensitization activities are essential for ensuring high treatment coverage

The CFIR construct *engaging* is defined as approaches to attracting and involving individuals in implementation, such as through social marketing or education campaigns (17). Adult participants across sites, including local leaders, identified specific leaders as key facilitators of effective implementation (Table 2). These leaders should be notified in advance of interventions taking place within their communities to gain their support and promote the intervention prior to implementation. Advance sensitization with information about the distribution time as well as potential intervention benefits and risks were identified as critical pieces of information that influence the implementation climate and, in effect, effective delivery of cMDA. Preferred engagement methods varied slightly across sites, however community members suggested that a variety of information-sharing mechanisms be utilized in advance of MDA to improve community member knowledge and buy-in (Table 3).

For peer review only

Table 3: Recommendations to optimize the implementation climate for newly launched cMDA

**Recommendation category**	**Benin**	**India**	**Malawi**
MDA distribution mode	Door-to-door distribution	Door-to-door distribution preferable; potential for 3-4 central distribution sites in some communities	Door-to-door distribution
Intervention cost/financial incentives for participation	Free, but need to address rumors about nefarious intentions behind free MDA distribution	Free treatment preferable to most participants; need to address fears of perceived poor-quality medications provided by government programs. Financial incentives should not be given for MDA participation, but incentives such as combs and soap were suggested	Free, but communities with past exposure to research studies might expect financial incentives for MDA participation
Community drug distributor preferences	Health workers (health facility workers or CDDs) who are familiar to community members	Trained health workers (nurses, doctors, ASHAs) who are familiar. Individuals without training should not be distributors	Health workers (including HSAs) who are familiar to community members. Volunteers are less respected and should not be distributors
Duration and time of distribution	Distribution over multiple days to accommodate different household schedules and reach the greatest number of people. Rainy season and market days should be avoided. Must consider work schedules and implement flexible distribution times	Distribution over multiple days. Evening or early morning preferred distribution time to accommodate work schedules	Distribution over multiple days to accommodate different household schedules and reach the greatest number of people
Key leaders to engage prior to cMDA	Village chiefs, religious leaders, and health workers	President and ward councilor of community (Panchayat), other health workers (Anganwadi workers), and teachers	Village chiefs, local leaders, religious leaders, local NGOs, HSAs, and teachers

For peer review only

Community education topics to engage MDA participants	Educate community about purpose and potential side effects of treatment	Educate community about purpose, advantages, and potential side effects of treatment, and proper dosage for different people (e.g., children, elders)	Educate community about purpose and potential side effects of treatment; sensitization must be done more than one day in advance to allow decision-making time
Mechanisms for engaging community members	Utilize radio, phones, community meetings, and word of mouth to share information. Ring gongs at distribution time	Utilize radio, loudspeaker announcements, flyers, health documentaries, TV news, community meetings (women's groups), and community dramas to share information. Beat drums at distribution time	Utilize radio, phones, loudspeaker announcements, dramas, community meetings, door-to-door outreach, to share information

For peer review only

DISCUSSION

This diagnostic analysis highlights opportunities and challenges of launching cMDA for STH that are shared across geographic areas as well as important differences between them. Our findings build upon the existing literature and demonstrate strong acceptability of cMDA for STH interruption, particularly as an alternative to school-based distribution to provide more equitable access to deworming treatment. When considering a transition from school-based distribution to cMDA, participants highlighted opportunities to integrate cMDA into existing community health programs, such as vaccination campaigns, and the importance of engaging clinically trained, trusted drug distributors to mitigate fears of adverse events and increase treatment coverage. Utilizing local CDDs from the same area or are directly known to the recipients, is associated with high MDA coverage in other settings. (21, 22) While familiarity was important to participants in this study, they also stressed comprehensive clinical training for CDDs as essential for fostering trust during cMDA. While participants identified potential benefits of launching cMDA, they also

For peer review only - http://bmjopen.bmj.com/site/about/guidelines.xhtml

noted key barriers that might limit implementation success. Similar to findings from studies exploring MDA barriers post-implementation, the primary barriers identified across sites included mistrust toward free drug distribution (especially those provided by community volunteers perceived to have no clinical training), fear of side effects, and limited perceived need for treatment without symptoms (12, 23-25). This information is essential for adapting interventions to fit the specific context and concerns of communities prior to making a significant change to public health programs, such as changing from school-based delivery to community-wide delivery of deworming medicines.

In this formative evaluation, we synthesized recommendations from community members to assist in intervention optimization at a site level, including preferred treatment time and distribution methods. Site specific preferences and recommendations for implementation varied to small degrees across settings, including preferred distribution times, campaign duration, location, distributor qualifications, and procedures for engaging leaders and community participants. Other studies have found that proactively identifying specific times when individuals are generally available to receive treatment is an essential facilitator of effective campaign delivery (26-28), and when not completed can increase frustration with community-based volunteer distributors (23) and the MDA campaign itself (29). Where trial timeline and funding allow, formative evaluations such as this may facilitate proactive identification of potential barriers and of implementation processes to optimize community acceptability and adapt intervention delivery as needed. When timelines or funding are limited, like during cMDA implemented by national NTD programs in limited resources settings, brief surveys prior to MDA and interim analyses and may be conducted to tailor MDA implementation.

Emerging themes from community member, local leader, CDD, and health worker FGDs were highly consistent. However, CDDs and health workers noted unique facilitators and barriers affecting their work, including time for training, resources in the field, and timely compensation, similar feedback from implementers in other settings (24). CDDs, health workers, and community leaders also uniquely stressed

For peer review only - http://bmjopen.bmj.com/site/about/guidelines.xhtml

the importance of packaging WASH interventions with MDA for STH elimination. These sentiments reflect advanced knowledge of STH transmission but may also reflect doubt that transmission interruption programs predicated on broadly delivered MDA will interrupt transmission without also improving hygiene infrastructure. In fact, most of the benefits attributed to cMDA amongst these cadres was driven by beliefs that MDA will be more acceptable when delivered in the community, as opposed to its potential for interrupt transmission. Although evidence regarding the effectiveness of WASH on STH transmission interruption is still weak, it is also important to monitor how implementer and community enthusiasm for cMDA changes over time, potentially driven by the absence of WASH interventions (30).

Participants across all geographic areas noted that myths and rumors could pose serious challenges to cMDA delivery, adding further to the literature documenting this obstacle in other settings (10, 24, 31, 32). Children in Benin and Malawi noted specific concerns about side effects of deworming treatment circulating within schools prior to and post-MDA campaigns. While side effects for albendazole are typically quite mild, albendazole is often co-administered to children with praziquantel as preventative chemotherapy for schistosomiasis. Praziquantel can cause relatively more severe side effects, including diarrhea and vomiting (33). Thus, prior experiences with MDA campaigns including other treatments might influence future perceptions about albendazole specifically or MDA generally (34). Evidence suggests that effective health workers can overcome these individual-level perceptions (29); addressing myths and rumors will require targeted and pro-active community sensitization and CDD training that openly discuss local myths and rumors pre-intervention.

Mistrust toward public health campaigns and government-run health programs was prevalent across settings. These concerns were driven by prior negative experiences with medical interventions and programs in which limited information was provided in advance of treatment, parents were minimally engaged in school-based MDA, and there were perceived concerns about drug quality. Potential strategies for overcoming these barriers include engaging local leaders (27, 31, 35), targeted education campaigns

For peer review only

For peer review only - http://bmjopen.bmj.com/site/about/guidelines.xhtml

(24, 36, 37), community mobilization (35, 38, 39), and engagement of trusted, trained personnel to administer preventive treatment.

During the design of our initial question guide (Appendix 1), we drew from selected constructs across all CFIR domains. However, during data analysis no constructs within the outer setting emerged as major facilitators or barriers to launch of cMDA campaigns. The outer setting domain is comprised of constructs representing external influences on implementation and, perhaps because these data were collected at the community-level, respondents were more focused on individuals involved in implementation and implementation processes (17). Additionally, while CFIR constructs should ideally be coupled to specific targeted outcomes, the formative nature of this study precludes linkage of implementation determinants to outcomes (18).

Conclusion

This study supports that cMDA, particularly as an alternative to school-based MDA, is generally acceptable across heterogenous settings and builds upon the existing literature exploring facilitators and barriers of launching and implementing cMDA. Community engagement including STH education, understanding preferred distribution times and methods, involvement of local leaders and familiar health workers or CDDs are critical for implementation success. Potential barriers, including mistrust of free drug distribution, fear of side effects, and limited perceived need of treatment can be addressed through community sensitization and engagement of local leaders and trusted health workers. Formative research exploring attitudes and community-derived recommendations should be conducted when possible to improve community acceptability of new interventions.

Author contributions

ARM, JLW, MCGC, SSRA, KK, and MI conceived of the initial study design. EA, RR, SPK, FC, CIT, PN, MI, KK, KA, AT, and YJ were involved in data collection and data processing. AR managed the data. AR, ABE, SL,

For peer review only - http://bmjopen.bmj.com/site/about/guidelines.xhtml

and ARM contributed to data analysis. SL, EA, and AM led the writing of the paper. All authors read and approved the final version of the manuscript.

Competing Interests

None declared.

Funding statement

The DeWorm3 study was funded by the Bill & Melinda Gates Foundation (https://www.gatesfoundation.org/) and (grant number OPP1129535). The funders had no role in study design, data collection and analysis, decision to publish, or preparation of the manuscript.

Data availability

Data are available from the DeWorm3 Institutional Data Access Committee (contact dw3data@uw.edu) for researchers who meet criteria for data access.

Acknowledgements

The authors wish to thank all of the study participants, communities and community leaders, national NTD program staff and other local, regional and national partners who have participated or supported the DeWorm3 study. We also would like to acknowledge the work of all members of the DeWorm3 study teams and affiliated institutions. We give special thanks to Rachel Geyer, Elodie Yard, and Mira Emmanuel-Fabula for contributions to data collection, management, or analysis. We thank Rajeshkumar Rajendran, Surya Kulasekaran and Chinnaduraipandi Paulsamy for their support in data collection in the field sites in India. We also thank Adolphe Kpatchavi, Roch Houngnihan and Abdoulaye Benon Monra for their support during the training of surveyors in the Benin site.

For peer review only

References

1. Hotez PJ, Molyneux DH, Fenwick A, Kumaresan J, Sachs SE, Sachs JD, et al. Control of neglected tropical diseases. New England Journal of Medicine. 2007;357(10):1018-27.

2. Jamison D, Breman J, Measham A, Alleyne G, Claeson M, Evans D, et al. Helminth Infections: Soil-transmitted Helminth Infections and Schistosomiasis--Disease Control Priorities in Developing Countries. 2006.

3. (WHO) WHO. Guideline on when to start antiretroviral therapy and on pre-exposure prophylaxis for HIV. 2015.

4. Brooker SJ, Nikolay B, Balabanova D, Pullan RL. Global feasibility assessment of interrupting the transmission of soil-transmitted helminths: a statistical modelling study. The Lancet infectious diseases. 2015;15(8):941-50.

5. Pullan RL, Halliday KE, Oswald WE, Mcharo C, Beaumont E, Kepha S, et al. Effects, equity, and cost of school-based and community-wide treatment strategies for soil-transmitted helminths in Kenya: a cluster-randomised controlled trial. The Lancet. 2019;393(10185):2039-50.

6. Lo NC, Bogoch II, Blackburn BG, Raso G, N'Goran EK, Coulibaly JT, et al. Comparison of community-wide, integrated mass drug administration strategies for schistosomiasis and soil-transmitted helminthiasis: a cost-effectiveness modelling study. The Lancet Global Health. 2015;3(10):e629-e38.

7. Farrell SH, Truscott JE, Anderson RM. The importance of patient compliance in repeated rounds of mass drug administration (MDA) for the elimination of intestinal helminth transmission. Parasites & vectors. 2017;10(1):291.

8. Shuford KV, Turner HC, Anderson RM. Compliance with anthelmintic treatment in the neglected tropical diseases control programmes: a systematic review. Parasites & vectors. 2016;9(1):29.

For peer review only - http://bmjopen.bmj.com/site/about/guidelines.xhtml

9. Yajima A, Mikhailov A, Mbabazi PS, Gabrielli AF, Minchiotti S, Montresor A, et al. Preventive Chemotherapy and Transmission Control (PCT) databank: a tool for planning, implementation and monitoring of integrated preventive chemotherapy for control of neglected tropical diseases. Transactions of the Royal Society of Tropical Medicine and Hygiene. 2012;106(4):215-22.

10. Krentel A, Fischer PU, Weil GJ. A review of factors that influence individual compliance with mass drug administration for elimination of lymphatic filariasis. PLoS Negl Trop Dis. 2013;7(11):e2447.

11. Gyapong JO, Owusu IO, Vroom FBd-C, Mensah EO, Gyapong M. Elimination of lymphatic filariasis: current perspectives on mass drug administration. Research and reports in tropical medicine. 2018;9:25.

12. Silumbwe A, Halwindi H, Zulu JM. How community engagement strategies shape participation in mass drug administration programmes for lymphatic filariasis: The case of Luangwa District, Zambia. PLoS Negl Trop Dis. 2019;13(11):e0007861.

13. Lugtenberg M, Burgers J, Westert G. Effects of evidence-based clinical practice guidelines on quality of care: a systematic review. BMJ Quality & Safety. 2009;18(5):385-92.

14. Ásbjörnsdóttir KH, Ajjampur SSR, Anderson RM, Bailey R, Gardiner I, Halliday KE, et al. Assessing the feasibility of interrupting the transmission of soil-transmitted helminths through mass drug administration: The DeWorm3 cluster randomized trial protocol. PLoS neglected tropical diseases. 2018;12(1).

15. Curran GM, Bauer M, Mittman B, Pyne JM, Stetler C. Effectiveness-implementation hybrid designs: combining elements of clinical effectiveness and implementation research to enhance public health impact. Medical care. 2012;50(3):217.

16. Means AR, Ajjampur SS, Bailey R, Galactionova K, Gwayi-Chore M-C, Halliday K, et al. Evaluating the sustainability, scalability, and replicability of an STH transmission interruption intervention: The DeWorm3 implementation science protocol. PLoS neglected tropical diseases. 2018;12(1):e0005988.

For peer review only

For peer review only - http://bmjopen.bmj.com/site/about/guidelines.xhtml

17. Damschroder LJ, Aron DC, Keith RE, Kirsh SR, Alexander JA, Lowery JC. Fostering implementation of health services research findings into practice: a consolidated framework for advancing implementation science. Implement Sci. 2009;4:50.

18. Kirk MA, Kelley C, Yankey N, Birken SA, Abadie B, Damschroder L. A systematic review of the use of the consolidated framework for implementation research. Implementation Science. 2015;11(1):72.

19. Cassell C, Symon G. Essential guide to qualitative methods in organizational research: Sage; 2004.

20. Nowell LS, Norris JM, White DE, Moules NJ. Thematic analysis: Striving to meet the trustworthiness criteria. International Journal of Qualitative Methods. 2017;16(1):1609406917733847.

21. Babu BV, Satyanarayana K. Factors responsible for coverage and compliance in mass drug administration during the programme to eliminate lymphatic filariasis in the East Godavari District, South India. Trop Doct. 2003;33(2):79-82.

22. Gunawardena S, Ismail M, Bradley M, Karunaweera N. Factors influencing drug compliance in the mass drug administration programme against filariasis in the Western province of Sri Lanka. Transactions of the Royal Society of Tropical Medicine and Hygiene. 2007;101(5):445-53.

23. Ahorlu CSK, Koka E, Adu-Amankwah S, Otchere J, de Souza DK. Community perspectives on persistent transmission of lymphatic filariasis in three hotspot districts in Ghana after 15 rounds of mass drug administration: a qualitative assessment. BMC public health. 2018;18(1):238.

24. Legge H, Kepha S, Prochazka M, Halliday K, Pullan R, Gwayi-Chore MC, et al. Implementer and recipient perspectives of community-wide mass drug administration for soil-transmitted helminths in Kwale County, Kenya. PLoS Negl Trop Dis. 2020;14(4):e0008258.

25. Babu BV, Mishra S. Mass drug administration under the programme to eliminate lymphatic filariasis in Orissa, India: a mixed-methods study to identify factors associated with compliance and non-compliance. Transactions of the Royal Society of Tropical Medicine and Hygiene. 2008;102(12):1207-13.

For peer review only

26. Dial NJ, Ceesay SJ, Gosling RD, D'Alessandro U, Baltzell KA. A qualitative study to assess community barriers to malaria mass drug administration trials in The Gambia. Malaria Journal. 2014;13(1):47.

27. Wanzira H, Naiga S, Mulebeke R, Bukenya F, Nabukenya M, Omoding O, et al. Community facilitators and barriers to a successful implementation of mass drug administration and indoor residual spraying for malaria prevention in Uganda: a qualitative study. Malar J. 2018;17(1):474.

28. Lahariya C, Mishra A. Strengthening of mass drug administration implementation is required to eliminate lymphatic filariasis from India: an evaluation study. J Vector Borne Dis. 2008;45(4):313-20.

29. Kisoka WJ, Simonsen PE, Malecela MN, Tersbøl BP, Mushi DL, Meyrowitsch DW. Factors influencing drug uptake during mass drug administration for control of lymphatic filariasis in rural and urban Tanzania. PLoS One. 2014;9(10):e109316.

30. Nery SV, Pickering AJ, Abate E, Asmare A, Barrett L, Benjamin-Chung J, et al. The role of water, sanitation and hygiene interventions in reducing soil-transmitted helminths: interpreting the evidence and identifying next steps. Parasites & vectors. 2019;12(1):273.

31. Gyapong JO, Owusu IO, da-Costa Vroom FB, Mensah EO, Gyapong M. Elimination of lymphatic filariasis: current perspectives on mass drug administration. Res Rep Trop Med. 2018;9:25-33.

32. Kisoka WJ, Tersbol BP, Meyrowitsch DW, Simonsen PE, Mushi DL. Community Members' Perceptions of Mass Drug Administration for Control of Lymphatic Filariasis in Rural and Urban Tanzania. J Biosoc Sci. 2016;48(1):94-112.

33. Sousa-Figueiredo JC, Pleasant J, Day M, Betson M, Rollinson D, Montresor A, et al. Treatment of intestinal schistosomiasis in Ugandan preschool children: best diagnosis, treatment efficacy and side-effects, and an extended praziquantel dosing pole. International Health. 2010;2(2):103-13.

For peer review only

For peer review only - http://bmjopen.bmj.com/site/about/guidelines.xhtml

34. Musuva RM, Matey E, Masaku J, Odhiambo G, Mwende F, Thuita I, et al. Lessons from implementing mass drug administration for soil transmitted helminths among pre-school aged children during school based deworming program at the Kenyan coast. BMC public health. 2017;17(1):575.

35. Omedo MO, Matey EJ, Awiti A, Ogutu M, Alaii J, Karanja DM, et al. Community health workers' experiences and perspectives on mass drug administration for schistosomiasis control in western Kenya: the SCORE Project. The American journal of tropical medicine and hygiene. 2012;87(6):1065-72.

36. Cantey PT, Rout J, Rao G, Williamson J, Fox LM. Increasing compliance with mass drug administration programs for lymphatic filariasis in India through education and lymphedema management programs. PLoS Negl Trop Dis. 2010;4(6):e728.

37. Garfield RM, Vermund SH. Health education and community participation in mass drug administration for malaria in Nicaragua. Social Science & Medicine. 1986;22(8):869-77.

38. De Martin S, Von Seidlein L, Deen JL, Pinder M, Walraven G, Greenwood B. Community perceptions of a mass administration of an antimalarial drug combination in The Gambia. Tropical Medicine & International Health. 2001;6(6):442-8.

39. Mulugeta A, Gebregergs GB, Asfaw S, Yemane D, Mitiku M, Meresa B, et al. Coverage, social mobilization and challenges of mass Zithromax administration campaign in South and South East zones of Tigray, Northern Ethiopia: A cross sectional study. PLoS neglected tropical diseases. 2018;12(2):e0006288.

For peer review only

For peer review only - http://bmjopen.bmj.com/site/about/guidelines.xhtml

Appendix 1. Qualitative interview guides

Interview guide #1: Health Centre & DeWorm3 Supervisory Teams

	Question
**1**	What kind of information or evidence are you aware of that shows whether or not community-wide MDA for interrupting transmission of STH will work in your setting?<br><br>*Follow-up questions:*<br>• *Is this evidence strong or weak?*<br>• *What evidence are you aware of from your own research? Practice guidelines? Published literature? Other settings?*<br>• *How does this knowledge affect your perception of the intervention?*
**2**	What advantages does community-wide MDA for STH have compared to school-age targeted programs? What disadvantages?
**3**	When delivering community-wide MDA for STH, what local adaptations should be made so that the intervention is effective?<br><br>*Follow-up questions:*<br>• *What aspects of the intervention should not be adapted?*<br>• *Do you think you will be able to make these adaptations? Why or why not?*
**4**	How complicated is delivery of the entire community-wide MDA for STH intervention? Please consider the following aspects of the intervention: duration of an MDA round, target population, and number of steps and sub-activities involved to fully implement.
**5**	What supportive materials or tools are needed to ensure effective implementation of community-wide MDA for STH?
**6**	What community-wide MDA for STH activities are built upon the previous LF MDA program infrastructure and experiences?<br><br>*Follow-up questions:*<br>• If none, what LF program activities could be leveraged for community-wide MDA for STH?
**7**	From your perspective, what are unique costs of implementing community-wide MDA for STH, relative to the standard of care of school-age targeted MDA?
**8**	What barriers might community members face in participating in community-wide MDA for STH?<br><br>*Follow-up question:*<br>• *What could be done to overcome these barriers?*
**9**	What kinds of infrastructure changes to the health system will be needed to accommodate community-wide MDA for STH?<br><br>*Follow-up questions:*

	Question
	• *If community-wide MDA for STH is scaled-up after the DeWorm3 project, will there need to be any changes in formal policies? Changes in information systems or data reporting systems? Other?*<br>• *What kind of approvals do you think will be needed to transition the STH program entirely to community-wide MDA? Who will need to be involved?*<br>• *Can you describe the process that will be needed to make these changes?*
**10**	How accepting are your co-workers of implementing community-wide MDA for STH? Why?
**11**	Do you think community-wide MDA for STH could be integrated into routine MOH programs?<br>*Follow-up questions:*<br>• If yes, how? If no, why not?<br>• *What would the effects of integration on routine programs be?*
**12**	How important do you think it is to implement community-wide MDA for STH compared to the other health priorities?
**13**	Do you feel incentivized to ensure that the DeWorm3 trial is successful in interrupting transmission of STH? What is the incentive?
**14**	How supportive are government and non-governmental leadership of implementing community-wide MDA for STH?<br>*Follow-up question:*<br>• *How do you think it will affect uptake/implementation?*
**15**	What kind of relevant training will you have during this intervention? Do you feel the training will prepare you to carry out the roles and responsibilities expected of you? How so?<br>*Follow-up questions:*<br>• *What are the positive aspects of the training?*<br>• *What is missing?*<br>• *What kind of continued training is necessary?*
**16**	Do you think that community-wide MDA could interrupt STH transmission (i.e. stop the spread of intestinal worms) in your setting? Why or why not?
**17**	How confident are you that you will be able to successfully carry out your DeWorm3 related duties? What gives you that level of confidence (or lack of confidence)?
**18**	Can you describe your team's plan for implementing community-wide MDA for STH?<br>*Follow-up questions:*<br>• *Do you think everyone involved understands the plan well, or is it too complicated?*<br>• *What do you do if you have to modify or revise the plan due to challenges, errors, or mistakes?*
**19**	Who are the key influential individuals or organizations to get buy-in from during the DeWorm3 study?

For peer review only

	Question
**20**	How should community members be informed about community-wide MDA or other DeWorm3 interventions before they occur?
**21**	What are some strategies to achieve high MDA treatment coverage?
**22**	Is there any other information that you would like to share about community-wide MDA today?

Interview Guide #2: Community Drug Distributors & Community Health Workers

#	Question
**1**	What advantages does community-wide MDA for STH have compared to school-age targeted programs? What disadvantages?
**2**	When delivering community-wide MDA for STH, what local adaptations should be made so that the intervention is effective?<br><br>*Follow-up questions:*<br>• What aspects of the intervention should not be adapted?<br>• Do you think you will be able to make these adaptations? Why or why not?
**3**	How complicated is delivery of the entire community-wide MDA for STH intervention? Please consider the following aspects of the intervention: duration of an MDA round, target population, and number of steps and sub-activities involved to fully implement.
**4**	What supportive materials or tools are needed to ensure effective implementation of community-wide MDA for STH?
**5**	From your perspective, what are unique costs of implementing community-wide MDA for STH, relative to the standard of care of school-age targeted MDA?
**6**	What barriers might community members face in participating in community-wide MDA for STH?<br><br>*Follow-up question:*<br>• *What could be done to overcome these barriers?*
**10**	How accepting are your co-workers of implementing community-wide MDA for STH? Why?
**11**	Do you think community-wide MDA for STH could be integrated into routine MOH programs?<br><br>*Follow-up questions:*<br>• *If yes, how? If no, why not?*<br>• *What would the effects of integration on routine programs be?*
**12**	How important do you think it is to implement community-wide MDA for STH compared to the other health priorities?
**13**	Do you feel incentivized to ensure that the DeWorm3 trial is successful in interrupting transmission of STH? What is the incentive?
**14**	How supportive are government and non-governmental leadership of implementing community-wide MDA for STH?

#	Question
	Follow-up question:<br>• *How do you think it will affect uptake/implementation?*
**15**	What kind of relevant training will you have during this intervention? Do you feel the training will prepare you to carry out the roles and responsibilities expected of you? How so?<br><br>*Follow-up questions:*<br>• *What are the positive aspects of the training?*<br>• *What is missing?*<br>• *What kind of continued training is necessary?*
**16**	Do you think that community-wide MDA could interrupt STH transmission (i.e. stop the spread of intestinal worms) in your setting? Why or why not?
**17**	How confident are you that you will be able to successfully carry out your DeWorm3 related duties? What gives you that level of confidence (or lack of confidence)?
**18**	Can you describe your team's plan for implementing community-wide MDA for STH?<br><br>*Follow-up questions:*<br>• *Do you think everyone involved understands the plan well, or is it too complicated?*<br>• *What do you do if you have to modify or revise the plan due to challenges, errors, or mistakes?*
**19**	Who are the key influential individuals or organizations to get buy-in from during the DeWorm3 study?
**20**	How should community members be informed about community-wide MDA or other DeWorm3 interventions before they occur?
**21**	What are some strategies to achieve high MDA treatment coverage?
**22**	Is there any other information that you would like to share about community-wide MDA today?

Interview Guide #3: Community members

#	Question
**1**	What advantages does community-wide MDA for STH have compared to school-age targeted programs?<br><br>*Follow-up question:*<br>• *What disadvantages?*
**2**	When delivering community-wide MDA for STH, what local adaptations should be made so that the intervention is effective?<br><br>*Follow-up question:*<br>• *What aspects of the intervention should not be adapted?*

#	Question
**3**	What barriers might community members face in participating in community-wide MDA for STH?<br><br>*Follow-up question:*<br>• *What could be done to overcome these barriers?*
**4**	Do you remember the lymphatic filariasis (LF) MDA programs that used to occur in this area? What do you remember about them?<br><br>*Follow-up questions:*<br>• *What could be done to overcome these barriers?*<br>• *Did you participate in those treatment days by swallowing the medicines given to you? Why or why not?*<br>• *Has that affected your interest in participating in the current mass treatment days?*
**5**	Do you think that community-wide MDA could interrupt STH transmission (i.e. stop the spread of intestinal worms) in your setting? Why or why not?
**6**	Who are the key influential individuals or organizations to get buy-in from during the DeWorm3 study?<br><br>*Follow-up questions:*<br>• *Can you provide specific examples of why you think their buy-in is important?*
**7**	How should community members be informed about community-wide MDA or other DeWorm3 interventions before they occur?
**8**	What are some strategies to achieve high MDA treatment coverage?
**9**	Is there any other information that you would like to share about community-wide MDA today?

review only

BMJ Open

"It depends on how you tell": A qualitative diagnostic analysis of the implementation climate for community-wide mass drug administration for soil-transmitted helminths

Journal:	*BMJ Open*
Manuscript ID	bmjopen-2022-061682.R1
Article Type:	Original research
Date Submitted by the Author:	11-May-2022
Complete List of Authors:	Avokpaho, Euripide; Institut de Recherche Clinique du Benin<br>Lawrence, Sarah; University of Washington, Pediatrics<br>Roll, Amy; University of Washington, Department of Global Health<br>Titus, Angelin; Christian Medical College Vellore<br>Jacob, Yesudoss; Christian Medical College Vellore<br>PUTHUPALAYAM KALIAPPAN, SARAVANAKUMAR; Christian Medical College Vellore, Division of Gastrointestinal Sciences<br>Gwayi-Chore, Marie Claire; University of Washington, Department of Global Health; The DeWorm3 Project<br>Chabi, Félicien; Institut de Recherche Clinique du Bénin<br>Togbevi, Comlanvi; Institut de Recherche Clinique du Bénin<br>Elijan, Abiguel Belou; Institut de Recherche Clinique du Bénin<br>Nindi, Providence; Blantyre Institute for Community Outreach<br>Walson, Judd L; University of Washington, Department of Global Health; The DeWorm3 Project<br>Ajjampur, Sitara; Christian Medical College Vellore, The Wellcome Trust Resaearch Laboratory, Division of Gastrointestinal Sciences<br>Ibikounle, Moudachirou; Institut de Recherche Clinique du Benin; Universite d'Abomey-Calavi, Centre de Recherche pour la lutte contre les Maladies Infectieuses Tropicales (CReMIT/TIDRC)<br>Kalua, Khumbo; Blantyre Institute for Community Outreach<br>Aruldas, Kumudha; Christian Medical College Vellore<br>Means, Arianna; University of Washington, Department of Global Health; The DeWorm3 Project
**Primary Subject Heading**:	Global health
Secondary Subject Heading:	Public health
Keywords:	Public health < INFECTIOUS DISEASES, Tropical medicine < INFECTIOUS DISEASES, Community child health < PAEDIATRICS, QUALITATIVE RESEARCH

SCHOLARONE™
Manuscripts

I, the Submitting Author has the right to grant and does grant on behalf of all authors of the Work (as defined in the below author licence), an exclusive licence and/or a non-exclusive licence for contributions from authors who are: i) UK Crown employees; ii) where BMJ has agreed a CC-BY licence shall apply, and/or iii) in accordance with the terms applicable for US Federal Government officers or employees acting as part of their official duties; on a worldwide, perpetual, irrevocable, royalty-free basis to BMJ Publishing Group Ltd ("BMJ") its licensees and where the relevant Journal is co-owned by BMJ to the co-owners of the Journal, to publish the Work in this journal and any other BMJ products and to exploit all rights, as set out in our licence.

The Submitting Author accepts and understands that any supply made under these terms is made by BMJ to the Submitting Author unless you are acting as an employee on behalf of your employer or a postgraduate student of an affiliated institution which is paying any applicable article publishing charge ("APC") for Open Access articles. Where the Submitting Author wishes to make the Work available on an Open Access basis (and intends to pay the relevant APC), the terms of reuse of such Open Access shall be governed by a Creative Commons licence – details of these licences and which Creative Commons licence will apply to this Work are set out in our licence referred to above.

Other than as permitted in any relevant BMJ Author's Self Archiving Policies, I confirm this Work has not been accepted for publication elsewhere, is not being considered for publication elsewhere and does not duplicate material already published. I confirm all authors consent to publication of this Work and authorise the granting of this licence.

For peer review only

It depends on how you tell: A qualitative diagnostic analysis of the implementation climate for community-wide mass drug administration for soil-transmitted helminths

Euripide Avokpaho[1†], Sarah Lawrence[2†], Amy Roll[2,3], Angelin Titus[4], Yesudoss Jacob[4], Saravanakumar Puthupalayam Kaliappan[4], Marie Claire Gwayi-Chore[2,3], Félicien Chabi[1], Comlanvi Innocent Togbevi[1], Abiguel Belou Elijan[1], Providence Nindi[5], Judd L Walson[2], Sitara Swarna Rao Ajjampur[4], Moudachirou Ibikounlé[1,6], Khumbo Kalua[5], Kumudha Aruldas[4], Arianna Rubin Means[*2,3]

† shared first authorship

[1] Institut de Recherche Clinique du Bénin, Abomey-Calavi, Bénin

[2] Department of Global Health, University of Washington, Seattle, WA, USA

[3] The DeWorm3 Project, Seattle, Washington, USA

[4] The Wellcome Trust Research Laboratory, Division of Gastrointestinal Sciences, Christian Medical College, Vellore, India

[5] Blantyre Institute for Community Outreach, Blantyre, Malawi

[6] Centre de Recherche pour la lutte contre les Maladies Infectieuses Tropicales (CReMIT/TIDRC), Université d'Abomey-Calavi, Benin

* Corresponding author

Email: aerubin@uw.edu

Word count: 5493

For peer review only

I, the Submitting Author has the right to grant and does grant on behalf of all authors of the Work (as defined in the below author licence), an exclusive licence and/or a non-exclusive licence for contributions from authors who are: i) UK Crown employees; ii) where BMJ has agreed a CC-BY licence shall apply, and/or iii) in accordance with the terms applicable for US Federal Government officers or employees acting as part of their official duties; on a worldwide, perpetual, irrevocable, royalty-free basis to BMJ Publishing Group Ltd ("BMJ") its licensees and where the relevant Journal is co-owned by BMJ to the co-owners of the Journal, to publish the Work in BMJ Global Health and any other BMJ products and to exploit all rights, as set out in our licence.

The Submitting Author accepts and understands that any supply made under these terms is made by BMJ to the Submitting Author unless you are acting as an employee on behalf of your employer or a postgraduate student of an affiliated institution which is paying any applicable article publishing charge ("APC") for Open Access articles. Where the Submitting Author wishes to make the Work available on an Open Access basis (and intends to pay the relevant APC), the terms of reuse of such Open Access shall be governed by a Creative Commons licence – details of these licences and which Creative Commons licence will apply to this Work are set out in our licence referred to above.

For peer review only

ABSTRACT

Objectives: Current soil-transmitted helminth (STH) morbidity control guidelines primarily target deworming of pre-school and school-age children. Emerging evidence suggests that community-wide mass drug administration (cMDA) may interrupt STH transmission. However, the success of such programs depends upon achieving high treatment coverage and uptake. This formative analysis was conducted to evaluate the implementation climate for cMDA and to determine barriers and facilitators to launch.

Settings: Prior to the launch of a cMDA trial in Benin, India, and Malawi.

Participants: Community members (adult women and men, children, and local leaders), community drug distributors (CDDs), and health facility workers.

Design: We conducted 48 focus group discussions (FGDs) with community members, 13 FGDs with CDDs, and 5 FGDs with health facility workers in twelve randomly selected clusters across the three study countries. We utilized the Consolidated Framework for Implementation Research (CFIR) to guide the design of the interview guide and thematic analysis.

Results: Across all three sites, aspects of the implementation climate that were facilitators to cMDA launch included: high community member demand for cMDA, integration of cMDA into existing vaccination campaigns and/or health services, and engagement with familiar health workers. Barriers to launching cMDA included mistrust toward medical interventions, fear of side effects, and limited perceived need for interrupting STH transmission. We include specific recommendations from community members regarding cMDA distribution sites, personnel requirements, delivery timing and incentives, leaders to engage, and methods for mobilizing participants.

Conclusions: Prior to launching the cMDA program as an alternative to school-based MDA, cMDA was found to be generally acceptable across diverse geographic and demographic settings. Community

For peer review only

For peer review only - http://bmjopen.bmj.com/site/about/guidelines.xhtml

members, CDDs, and health workers felt that engaging communities and tailoring programs to the local context are critical for success. Potential barriers may be mitigated by identifying local concerns and addressing them via targeted community sensitization prior to implementation.

Strengths and limitations of this study

- This study conducted focus group discussions across three diverse settings, creating the opportunity to understand similarities and differences in the implementation climate for community -wide mass drug administration (cMDA) and STH transmission interruption.
- Some participants may have heard about the intervention before participating in focus-group discussions (FGDs), which may pose threats to social desirability and response biases.
- Although a large number of FGDs were conducted across heterogeneous settings, the generalizability of study findings may not be translatable to other STH-endemic areas.

INTRODUCTION

Neglected tropical diseases (NTDs) affect some of the world's most impoverished populations and contribute to a variety of morbidities that exacerbate existing health and economic inequities (1). Infections with one group of NTD, soil-transmitted helminths (STH), are associated with anemia, cognitive development delay, growth stunting, pre-term birth, and other adverse outcomes (2). World Health Organization (WHO) guidelines recommend control of STH morbidities via annual or bi-annual deworming of pre-school and school-age children and other high-risk groups, including pregnant women and adolescent girls (3). However, in many settings, the current STH strategy would likely need to be continued

For peer review only - http://bmjopen.bmj.com/site/about/guidelines.xhtml

until significant economic development and universal water, sanitation and hygiene (WASH) access are broadly available to stop transmission of STH (4). Emerging evidence suggests that it may be possible to interrupt transmission of STH by expanding deworming to treat individuals of all ages via community-wide MDA (cMDA) (5, 6).

The current standard of care for STH is school-based MDA to reach pre-school and school-age children, and many school-based deworming programs have been successfully implemented for decades. Transitioning from school-based MDA to cMDA for community-level STH transmission interruption will require adapting long withstanding programs or designing new platforms for reaching much larger target populations. These transitions should be approached carefully, as they will likely affect community member and health worker attitudes towards and engagement in new programs. The success of cMDA in interrupting transmission of STH is indeed predicated upon programs attaining high treatment coverage (drug receipt) and uptake (drug ingestion) (7, 8). Many STH-endemic communities have a long history of participating in other community-based mass distribution programs, including campaigns for lymphatic filariasis (LF), onchocerciasis, trachoma, malaria bed net distribution, and/or mass immunization programs such as polio campaigns (9). Factors that influence participation in mass campaigns include recipient trust in programs and efforts to tailor programs to local conditions (10). Negative campaign experiences or perceptions can compromise the success of future programs, particularly those requiring high coverage over multiple years to reach targeted transmission endpoints (11, 12).

Formative qualitative research can be used to understand community-member and implementer perceptions of past, ongoing, or prospective community-based campaigns. Diagnostic analyses, an application of formative evaluations, are particularly helpful in illuminating processes that can facilitate or impede implementation. Diagnostic analyses help to identify determinants of current practices, potential barriers and facilitators to implementing new interventions, and the perceived feasibility or utility of a new implementation strategy. This formative evidence can help researchers and implementers

For peer review only

understand potential implementation challenges and, ideally, address them prior to intervention launch (13). In this study, we perform a diagnostic analysis of the implementation climate to proactively identify factors influencing the launch of cMDA for STH transmission interruption, including (1) perceptions of current deworming practice, (2) potential barriers and facilitators to transitioning from school-based MDA to cMDA delivery, and (3) perceived effectiveness and need for cMDA (14).

METHODS

This analysis was conducted at the outset of the DeWorm3 Project, a large hybrid type I community cluster randomized trial in Benin, India, and Malawi (Table 1). Launched in 2017, the currently underway DeWorm3 Project aims to determine the feasibility of interrupting STH transmission using twice annual cMDA treating eligible individuals of all ages, relative to standard-of-care school-based MDA. More information about the DeWorm3 cluster randomized trial design has been described in detail elsewhere (15-17).

For peer review only - http://bmjopen.bmj.com/site/about/guidelines.xhtml

Table 1. Overview of study sites

	Benin	India	Malawi
**Site**	Commune of Comè	Vellore and Thiruvannamalai Districts, Tamil Nadu	Mangochi District
**Geographic area of site (km²)**	148	477	289
**Total number of households**	24,378	36,536	27,750
**Population size**	94,969	140,932	121,819
**Standard of care**	Annual school-based MDA targeting children 5-14 years of age	Biannual school-based MDA on National Deworming Days targeting children 1-19 years of age	Annual school-based MDA and Child Health Days targeting children 1-14 years of age
**cMDA workforce**	Community drug distributors (CDDs), coordinated by the Ministry of Health	CDDs and Accredited Social Health Activists (ASHA), women working as health educators and promoters in their communities	Community health workers (Health Surveillance Assistants) who also fill the rolls of CDDs, coordinating with teachers

Sampling strategy

Key stakeholders shaping the implementation climate for cMDA include community members and local health workers. Focus group discussions (FGDs) were conducted separately with groups of community members, including adult women and men (over 15 years), community leaders, and children (ages 12-15 years of age), local health workers, including CDDs, and Ministry of Health (MOH) health facility workers who often serve as CDD supervisors.

Prior to trial randomization (e.g., before designations of intervention or control clusters were made), four clusters were randomly selected in each site to conduct community-level FGDs. In each cluster, one FGD was conducted within each community member strata (four total), two FGDs among drug distributors, and one FGD among local MOH health facility workers. The sampling strategy for identifying and recruiting community members for FGDs within each cluster differed slightly by site (Table 2). In India, purposive sampling was employed, in which village leaders/influencers identified potential participants. In Malawi, community members were selected to participate via pseudo-randomization from a pool of individuals

For peer review only

who attended outreach meetings at the chiefs/headmen's residence. The first five randomly approached individuals from each demographic strata who agreed to participate were invited to attend FGDs within the next week (except children, for whom parents/caregivers were approached). In Benin, community members were selected from a randomly generated list of potential participants from a baseline census database. The research team contacted the household heads by telephone and invited a specific individual (woman, man, or child) to participate in an FGD. No more than one individual per household was selected to participate in an FGD in a given cluster. Transportation was offered to individuals who needed access to the FGD location. In Benin and India, local leaders were chosen using purposive quota sampling, during which DeWorm3 study teams invited key leaders in each selected cluster. Leaders differ setting by setting, wherein in some countries key leaders primarily include village chiefs while in other areas key leaders are primarily religious leaders. Purposive quota sampling was also used to invite CDDs and health workers from local health facilities located in each cluster to participate in FGDs.

For peer review only

Table 2: Sampling strategy by stakeholder group

Stakeholder	Targeted sample size (per FGD)	Sampling strategy
Community members		
Adult women (15+ years of age)	5-10	Purposive sampling (India)<br>Random sampling (Benin[1] and Malawi)
Adult men (15+ years of age)	5-10	
Local leaders	5-10	
Children (12-15 years of age)	5-10	
Health center staff and CDD supervisors	5-10	Purposive quota sampling
Community drug distributors (CDDs)	10-15	Purposive quota sampling

[1] Purposive quota sampling was used to sample local leaders in Benin and India

Data collection

This diagnostic analysis study design is informed by the Consolidated Framework for Implementation Research (CFIR), a meta-theoretical framework of 38 constructs that provides a typology of constructs for characterizing potential determinants (barriers and facilitators) to implementation from the perspective

For peer review only - http://bmjopen.bmj.com/site/about/guidelines.xhtml

of individuals involved in implementation (18). The CFIR has been used widely in low-and-middle-income countries to identify factors that could influence or are actively influencing successful implementation (19). CFIR constructs are organized according to five major domains influencing implementation and implementation effectiveness including (1) the intervention, (2) the inner setting, (3) the outer settings, (4) the individuals involved, and (5) the process for accomplishing the intervention. While the CFIR can be used at any stage of implementation, when applied pre-implementation, the CFIR can help proactively identify opportunities and challenges facing implementation and inform adaptations to implementation strategies for the local context (18, 20).

We drew upon the CFIR to inform the design of four semi-structured interview guides with a mix of respondent and informant style questions, tailored to each stakeholder group (one question guide was used for all adult community members). In this study, we identified *a priori* 23 CFIR constructs across all five domains that we hypothesized would influence the implementation climate for cMDA and which were appropriate for use during formative diagnostic research prior to implementation (Appendix 1) (20). The question guides were piloted and adapted slightly by changing word choice or sentence construction as necessary within each site to ensure that the questions were clear, meaningful, and culturally appropriate. Site adapted question guides were thereafter translated into local languages including Yao (Malawi) and Tamil (India), and the official language (French) in Benin. FGD facilitators in Benin adapted the French question guide to local languages, including Watchi and Pédah when necessary, during FGD facilitation.

All participants provided written consent prior to the start of the FGD. The parents or caregivers of participating children similarly provided written consent and children ages 12-15 also provided written assent. Consent and assent could also be provided by a thumbprint in the presence of a witness. FGDs were conducted in private locations with both a facilitator and notetaker present and all FGDs were audio-recorded with participant permission.

For peer review only

Analysis

Audio files were transcribed verbatim in the local or official language. For each transcript, two one-minute random spot checks were conducted on each audio file for quality assurance. All transcripts were then translated into English. All transcriptions and their translations were reviewed by a second individual fluent in both English and the local language for quality assurance. Transcripts were imported into ATLAS.ti version 8 (Scientific Software Development GmbH, Berlin, Germany), which was used to manage data analysis. Coders were based in each DeWorm3 site as well as at the central level (University of Washington, Seattle). For data collected in Benin and India, two primary coders were assigned to each transcript, with a third coder designated as the "tie-breaker." When possible, at least one coder was based at the site in which the data were collected, and the other coder was a member of the DeWorm3 central team. For data collected in Malawi, a single primary coder from the central level coded the data while a secondary coder at the site reviewed and validated the findings, due to coder availability. Each primary coder independently read and coded each transcript primarily using a deductive approach and a CFIR-based codebook. Coding teams from each country and the central level met via conference calls to iteratively refine code definitions and code inclusion/exclusion criteria until a final codebook was established. After a transcript was coded, the coders assigned to the transcript met via conference call for consensus meetings to discuss where applied codes diverged. When necessary, a third coder weighed in where consensus between primary coders was not reached. Data saturation was reached as no new themes emerged during iterative review of the collected data.

For peer review only

The final coded transcripts were used to create case memos that were grouped by stakeholder category and site. The case memos included a summary of how the code was applied for a given stakeholder group, a justification for the summary provided noting code patterns and latent messages, and specific quotes highlighting how the code was applied. The summaries, patterns, and themes from the coded transcripts

For peer review only - http://bmjopen.bmj.com/site/about/guidelines.xhtml

and case memos were used to guide thematic analysis, an analytical method that is useful for summarizing key features of large datasets using a clearly-structured approach (21, 22).

Patient and public involvement

Community members and health workers living in the sampled STH endemic areas were not involved in design, conduct, or reporting of this qualitative study. However, all feedback from community members was used to shape a subsequent community-based intervention within a larger clinical trial.

Ethical approval

This study has been reviewed and approved by the Institut de Recherche Clinique du Bénin (IRCB) through the National Ethics Committee for Health Research (002-2017/CNERS-MS) from the Ministry of Health in Benin, The London School of Hygiene and Tropical Medicine (12013), The College of Medicine Research Ethics Committee (P.04/17/2161) in Malawi, and Christian Medical College, Vellore, in India (10392). The study was also approved by The Human Subjects Division at the University of Washington (STUDY00000180).

RESULTS

In this study, 48 FGDs were conducted with community members – four FGDs (one per cluster) for each stakeholder group: adult women, adult men, children, and local leaders, totaling 16 in each site, 13 with CDDs (two each in Benin and Malawi, nine in India), and five with CDD supervisors (two in Benin and three in India).

Across FGDs and settings, key themes emerged within four CFIR domains and are presented accordingly below: intervention characteristics, inner settings, characteristics of individuals, and process. Factors positively influencing the implementation climate for cMDA across sites included community member demand for community-wide (versus school-based) MDA, integration of MDA into existing vaccination

For peer review only - http://bmjopen.bmj.com/site/about/guidelines.xhtml

campaigns and/or health services, and engagement with health workers (including trained CDDs) rather than community volunteers. Factors negatively affecting the implementation climate across sites included mistrust and resistance toward medical interventions, fear of side effects, and limited perceived need. Additional process recommendations emerged as key themes that varied slightly across sites and included suggestions regarding MDA distribution sites and distributors, treatment costs/financial incentives, engaging leaders, and engaging participants through sensitization and mobilization efforts.

Intervention characteristics

Relative advantage: cMDA is preferable to school-based MDA

The CFIR construct of *relative advantage* captures participant perceptions regarding the benefits of implementing one intervention compared to an alternative (18). Across community member and health worker/CDD groups and sites, participants identified a preference for cMDA as compared to school-based deworming programs for several reasons. Participants stressed that providing STH treatment to both children and adults is the only way to prevent STH reinfection.

Across stakeholder groups, participants also highlighted that children who were not enrolled in school would be able to receive treatment through cMDA. Adult community members in Benin and Malawi were particularly concerned that school-based MDA campaigns do not always provide parents with treatment information prior to distribution and often administer the medications without parental consent or trust. Additionally, they thought uptake would be improved if parents are involved in treatment administration.

> *It's better to go through the parents to reach the kids. Parents know how to approach their children, manage them and make them understand the benefit of the thing [medicine]. The child will easily take the medicine without any effect.* (Cluster 26, Women, Benin)

For peer review only - http://bmjopen.bmj.com/site/about/guidelines.xhtml

Across FGDs and sites, participants were enthusiastic that cMDA could interrupt STH transmission and increase parental engagement with the intervention, particularly to enable parental consent, and allow them to encourage and confirm their child's uptake.

Design quality & packaging: Door-to-door MDA delivery by highly trained, familiar distributors is preferred

Design quality describes stakeholder recommendations for how to bundle, present, and assemble the intervention (18). Across sites and stakeholder groups, campaigns that delivered services door-to-door were considered more desirable than those that used a central distribution site (Table 3). In India, community participants reflected upon past experiences with LF MDA campaigns that were door-to-door whereas participants in Benin reflected upon experiences with door-to-door vaccination campaigns and bed net distributions at local health centers. Long waiting times, associated with lost income and productivity, were identified as primary barriers to central distribution sites. One female participant in Benin reported she would not wait around all day for someone to distribute MDA but instead would just purchase the medications herself, given their low costs. Additionally, participants stressed that door-to-door campaigns improve equity by increasing the likelihood of reaching those unable to travel due to financial or physical barriers.

> *The HSAs should go door by door to give people the medicine as some people, for example old and crippled, may not be able to go and access the drugs. But if they go door by door, then everyone receives the drugs and not only those who walk.* (Cluster 21, Women, Malawi)

Community members and leaders across sites, participants preferred to receive treatment from individuals perceived to be health professionals, especially a familiar health worker or CDD, or someone working with a well-respected non-governmental organization (NGO). Participants believed that increased health worker engagement could alleviate community mistrust linked with fear of adverse events by medicalizing the distribution process and making community members feel safer, thereby

For peer review only

For peer review only - http://bmjopen.bmj.com/site/about/guidelines.xhtml

increase treatment coverage. Men in Malawi stressed that health volunteers are often poorly respected and mistrusted, while clinically-trained health professionals are typically more respected. Of paramount importance for adult community members and CDDs was that distributors are known members of the community.

> *When community members see new faces during a project, they tend to be resistant, so it is better to use people from the area and not strangers. If not, this may not be successful.* (Cluster 21, Local leader, Malawi)

> *But above all, it is necessary to involve health workers, who the population trusts....Many are afraid because they do not see us, they do not see the health workers on the ground.* (Health Center Staff, Benin)

In Benin, local leaders noted that when NGOs engage in cMDA, it is important they are well-respected and have well-recognized logos that community members are familiar with and trust based on their prior work. Regardless if cMDA is administered by a health professional, volunteer, or NGO, community members across sites noted that their willingness to participate in cMDA is driven by their perception that they have been fully and accurately informed about cMDA, and that they have had time to ask questions.

> *Even if it [deworming medication] is given for free, they will not eat it unless it has been explained and given. If they are told they will benefit...with awareness in the villages, they will eat it.* (Cluster 12, CDD, India)

Community members expressed that they wanted to be treated with dignity and that their participation in community-wide public health campaigns of any kind should not be taken for granted.

> *Whether they eat the tablet or not it depends on to what extent this information reaches the people. It depends on how you tell.* (Cluster 34, Men, India)

For peer review only

For peer review only - http://bmjopen.bmj.com/site/about/guidelines.xhtml

> *The messages about drugs should be given to us in good time and not just tell us like today that tomorrow we will have a drug administration activity. Many people need time to ask questions and clear their myths before they get treated. Some people tend to refuse medicine because of fear of side effects, so when you sensitize them for a long time, they tend to listen and at the end the program becomes successful. So avoid short notice messages, people are difficult. They need enough time to understand what is happening.* (Cluster 21, Men, Malawi)

In Malawi, local leaders reported that community members want to be followed up with after distribution to monitor for adverse events or continued engagement with distribution programs to foster trust in future campaigns. Without this, the leaders feared that negative rumors might proliferate, or communities might feel as though they only received treatment for research purposes, rather than for their wellbeing.

Intervention complexity: cMDA is complex, but still feasible to implement

The CFIR construct of *complexity* is defined as the perceived difficulty of implementing an intervention (18). Across groups, participants were concerned about the timing of cMDA, the distance to distribution sites if cMDA is centrally located as opposed to delivered door-to-door, and whether or not they would have sufficient notice about cMDA before the campaign begins. Many community members suggested optimal distribution times, which varied by site depending on common work schedules and holidays. Concerns regarding health worker/CDD knowledge and accommodation of community members' schedules were prevalent across FGDs but some participants stressed they would change their schedules to be present for distribution if informed by community leaders.

> *Even if someone has a plan to go to the field or to the market, three days before the distribution of the drug, they will cancel their plan and come and listen to what the village chief invited them to do...if everyone is not informed, it [MDA] cannot succeed*. (Cluster 10, Women, Benin)

For peer review only

Adults and children across sites recommended that distribution over multiple days within a community to reach the greatest number of people.

> *The period of drug administration should be long so that everyone is able to receive treatment. Some people may not be home during the time that you have set to administer the drugs and as such if done for maybe only a day, it means those people will not receive the drugs. But if it is for some more days then everyone will be treated.* (Cluster 21, Men, Malawi)

Inner setting

Implementation climate: Initial mistrust of MDA is likely, but demand and perceived need will counter this

The CFIR construct *implementation climate* captures comments related to the community member's receptivity to implementation, and the extent to which implementation is supported (18). The core component of implementation climate discussed across FGDs were factors that influence community member trust in treatment campaigns. Participants across the sites anticipated high levels of initial mistrust and potential resistance toward newly launched cMDA for STH. This initial mistrust is driven by personal and anecdotal evidence of adverse side-effects such as fatigue, stomachaches, and fever after previous school-based deworming MDA campaigns.

> *Other pupils received the medical treatment before they ate a meal, hence they vomited. So those that did not receive the medical treatment were afraid of vomiting too if they took the medicine.* (Cluster 19, Children, Malawi)

> *They will eat [medication] based on the trust. They will eat [medication] based on your approach, otherwise they may take and keep it aside somewhere....* (Cluster 17, Men, India)

In Malawi, limited follow-up by transitory MDA programs and research projects was also noted as fueling mistrust of community health programs. Similarly, in one CDD FGD in India, participants identified mistrust

For peer review only - http://bmjopen.bmj.com/site/about/guidelines.xhtml

of government programs as a potential barrier to MDA campaigns where medications are provided for free. CDDs explained that community members perceive government provided medications to be of poorer quality and therefore less effective with greater risks of side effects; therefore, those who can afford to purchase their own medications from pharmacies will often do so.

> *For people who can, they will get it [deworming medicine] from the medical shop. Whatever is given through the government they will keep it aside and they will not use it.* (Cluster 15, CDD, India)

In Benin, some participants from the men's, women's, and CDD FGDs shared concerns that drugs used in such campaigns might be given by Westerners with malintent.

> *The majority of the population does not understand. They think that the drugs are poisoned in order to reduce the African population.* (Cluster 10, Men, Benin)

Similarly, in Malawi, CDDs identified rumors and misinformation as major barriers to delivering cMDA with high coverage. Specific rumors include that stool collected for STH surveillance would be used for Satanist practices, rather than medical purposes, and that school-based deworming programs provide contraceptives to young children to reduce population growth.

While participants noted that mistrust and resistance might initially be high following a transition to cMDA for STH, there was still a strong perceived demand for deworming of all ages, and a sentiment that community sensitization could overcome these concerns.

> *...we are looking forward to this [community-based MDA] and we would like this to be a regular treatment. People are suffering from intestinal worms and only children receive the treatment. So this project [DeWorm3] will help all of us to receive treatment.* (Cluster 21, Men, Malawi)

Compatibility: Community-based MDA is highly compatible with existing health infrastructure

For peer review only

The CFIR construct *compatibility* is highly related to *implementation climate*, capturing the alignment between the innovation and existing values and priorities (18). Health facility workers and CDDs across sites noted cMDA should be integrated into existing community programs or, at a minimum, coordinate with ongoing community-based activities to improve treatment coverage and mitigate risks of conflict with ongoing local health programs. CDDs in India and community leaders in Benin identified community-based vitamin A and iron distribution and childhood vaccination campaigns as ideal programs to integrate with cMDA. In India, CDDs also suggested integrating cMDA with existing indoor residual spraying programs for vector control.

> *The voluntary workers who go house to house to spray mosquitoes, we can make use of them to give the tablet...Earlier they were going once a month or once a week, but now they go daily house to house. We can give through them.* (Cluster 17, CDD, India)

Available resources: Training, storage, and hygiene infrastructure are key resources for MDA implementation

The CFIR construct *available resources* describes the financial and material resources available (and desired) for implementation, including training and education, space, time, and money (18). Health facility workers and CDDs across sites stressed that existing resources may not be sufficient for delivery of cMDA. CDDs in particular were concerned about receiving adequate training and access to resources to take home for self-review. CDDs and health workers highlighted that they wanted more than a single one-day training prior to MDA, in order to provide adequate time to practice and apply skills in a training environment. Across sites, CDDs noted the importance of training before distribution.

Other key resources identified by CDDs and health workers in Benin included medication storage in the field, community education materials, shelter during inclement weather, as well as food, water, and financial incentives for CDDs. CDDs in Benin were particularly concerned with timely payment for their

For peer review only

For peer review only - http://bmjopen.bmj.com/site/about/guidelines.xhtml

work. In Malawi, local leaders noted that in the past villagers have felt burdened by volunteering for health program implementation without compensation. They also noted that villagers might expect payment for participating in MDA, given past experiences with research projects providing stipends. In India, health facility workers wanted to ensure they would have adequate staffing to assist during MDA.

Lastly, CDDs and health center personnel across sites, women in Benin, and leaders in India stressed that hygiene infrastructure needs to be improved and that investing in WASH as part of a broader STH elimination program might, as a result, increase treatment coverage of cMDA by demonstrating long-term investments in community well-being.

> *The rules of hygiene are very important, very, very important. Without it, we cannot right away start distributing the drugs and say that we want to completely eradicate the transmission of worms, impossible.* (CDD, Benin)

Characteristics of individuals

Knowledge & beliefs: Skepticism of the clinical rationale for MDA, fear of side effects, and limited perceived need for treatment will be barriers to cMDA

The *knowledge and beliefs* CFIR construct is defined as individuals' attitudes toward and value placed on implementation and their familiarity with related facts, truths, and principles (18). While participants strongly believed cMDA could eliminate STH transmission, some reservations about MDA rooted in knowledge and perceptions about deworming medications remain. For example, adult men and children in Benin and local leaders in Malawi raised concerns about the effects of treating people who may not be infected with STH.

> *When you get the drugs and you do not have the worms, the tablet can still damage your organs such as organs of digestion or breathing.* (Cluster 1, Children, Benin)

For peer review only

For peer review only - http://bmjopen.bmj.com/site/about/guidelines.xhtml

In India and Malawi, participants in the men's FGDs thought individuals who feel healthy might perceive themselves to be at low risk of STH and thus choose not to participate in cMDA. Women in Malawi reported this occurred during prior cMDA campaigns while women in India also noted individuals with limited literacy might not understand the need for treatment and be reluctant to participate in MDA campaigns.

Self-efficacy: Community members and CDDs will be key mobilizers for cMDA

The CFIR construct *self-efficacy* captures comments that reflect an individual's beliefs in their own abilities to achieve implementation goals (18). Women and community leaders in Benin identified themselves as important catalysts in influencing the acceptability of MDA by working together and influencing their social networks.

> *The process is simple as we have just understood, we will also explain to our brothers and sisters who will not accept that treatment is a good thing. We will tell them they should not be discouraged adding that there is good in it. It's up to us to explain to them.* (Cluster 1, Women, Benin)

Health facility workers and CDDs also identified themselves as important contributors to ensuring successful MDA implementation, given their existing relationships with communities.

> *We worked with them and they know us on the ground to be able to do the job, so there are no issues for community health workers.* (CDD, Benin)

Process

Engaging: Local leaders and sensitization activities are essential for ensuring high treatment coverage

For peer review only - http://bmjopen.bmj.com/site/about/guidelines.xhtml

The CFIR construct *engaging* is defined as approaches to attracting and involving individuals in implementation, such as through social marketing or education campaigns (18). Adult participants across sites, including local leaders, identified specific leaders as key facilitators of effective implementation (Table 2). These leaders should be notified in advance of interventions taking place within their communities to gain their support and promote the intervention prior to implementation. Advance sensitization with information about the distribution time as well as potential intervention benefits and risks were identified as critical pieces of information that influence the implementation climate and, in effect, effective delivery of cMDA. Preferred engagement methods varied slightly across sites, however community members suggested that a variety of information-sharing mechanisms be utilized in advance of MDA to improve community member knowledge and buy-in (Table 3).

Table 3: Recommendations to optimize the implementation climate for newly launched cMDA

Recommendation category	Benin	India	Malawi
MDA distribution mode	Door-to-door distribution	Door-to-door distribution preferable; potential for 3-4 central distribution sites in some communities	Door-to-door distribution
Intervention cost/financial incentives for participation	Free, but need to address rumors about nefarious intentions behind free MDA distribution	Free treatment preferable to most participants; need to address fears of perceived poor-quality medications provided by government programs. Financial incentives should not be given for MDA participation, but incentives such as combs and soap were suggested	Free, but communities with past exposure to research studies might expect financial incentives for MDA participation
Community drug distributor preferences	Health workers (health facility workers or CDDs) who are familiar to community members	Trained health workers (nurses, doctors, ASHAs) who are familiar. Individuals without training should not be distributors	Health workers (including HSAs) who are familiar to community members. Volunteers are less

For peer review only

For peer review only - http://bmjopen.bmj.com/site/about/guidelines.xhtml

			respected and should not be distributors
Duration and time of distribution	Distribution over multiple days to accommodate different household schedules and reach the greatest number of people. Rainy season and market days should be avoided. Must consider work schedules and implement flexible distribution times	Distribution over multiple days. Evening or early morning preferred distribution time to accommodate work schedules	Distribution over multiple days to accommodate different household schedules and reach the greatest number of people
Key leaders to engage prior to cMDA	Village chiefs, religious leaders, and health workers	President and ward councilor of community (Panchayat), other health workers (Anganwadi workers), and teachers	Village chiefs, local leaders, religious leaders, local NGOs, HSAs, and teachers
Community education topics to engage MDA participants	Educate community about purpose and potential side effects of treatment	Educate community about purpose, advantages, and potential side effects of treatment, and proper dosage for different people (e.g., children, elders)	Educate community about purpose and potential side effects of treatment; sensitization must be done more than one day in advance to allow decision-making time
Mechanisms for engaging community members	Utilize radio, phones, community meetings, and word of mouth to share information. Ring gongs at distribution time	Utilize radio, loudspeaker announcements, flyers, health documentaries, TV news, community meetings (women's groups), and community dramas to share information. Beat drums at distribution time	Utilize radio, phones, loudspeaker announcements, dramas, community meetings, door-to-door outreach, to share information

For peer review only

DISCUSSION

This diagnostic analysis highlights opportunities and challenges of launching cMDA for STH that are shared across geographic areas as well as important differences between them. Our findings build upon the

For peer review only - http://bmjopen.bmj.com/site/about/guidelines.xhtml

existing literature and demonstrate strong acceptability of cMDA for STH interruption, particularly as an alternative to school-based distribution to provide more equitable access to deworming treatment. When considering a transition from school-based distribution to cMDA, participants highlighted opportunities to integrate cMDA into existing community health programs, such as vaccination campaigns, and the importance of engaging clinically trained, trusted drug distributors to mitigate fears of adverse events and increase treatment coverage. Utilizing local CDDs from the same area or are directly known to the recipients, is associated with high MDA coverage in other settings. (23, 24) While familiarity was important to participants in this study, they also stressed comprehensive clinical training for CDDs as essential for fostering trust during cMDA. While participants identified potential benefits of launching cMDA, they also noted key barriers that might limit implementation success. Similar to findings from studies exploring MDA barriers post-implementation, the primary barriers identified across sites included mistrust toward free drug distribution (especially those provided by community volunteers perceived to have no clinical training), fear of side effects, and limited perceived need for treatment without symptoms (12, 25-27). This information is essential for adapting interventions to fit the specific context and concerns of communities prior to making a significant change to public health programs, such as changing from school-based delivery to community-wide delivery of deworming medicines.

In this formative evaluation, we synthesized recommendations from community members to assist in intervention optimization at a site level, including preferred treatment time and distribution methods. Site specific preferences and recommendations for implementation varied to small degrees across settings, including preferred distribution times, campaign duration, location, distributor qualifications, and procedures for engaging leaders and community participants. Other studies have found that proactively identifying specific times when individuals are generally available to receive treatment is an essential facilitator of effective campaign delivery (28-30), and when not completed can increase frustration with community-based volunteer distributors (25) and the MDA campaign itself (31). Where

For peer review only

trial timeline and funding allow, formative evaluations such as this may facilitate proactive identification of potential barriers and of implementation processes to optimize community acceptability and adapt intervention delivery as needed. When timelines or funding are limited, like during cMDA implemented by national NTD programs in limited resources settings, brief surveys prior to MDA and interim analyses and may be conducted to tailor MDA implementation.

Emerging themes from community member, local leader, CDD, and health worker FGDs were highly consistent. However, CDDs and health workers noted unique facilitators and barriers affecting their work, including time for training, resources in the field, and timely compensation, similar feedback from implementers in other settings (26). CDDs, health workers, and community leaders also uniquely stressed the importance of packaging WASH interventions with MDA for STH elimination. These sentiments reflect advanced knowledge of STH transmission but may also reflect doubt that transmission interruption programs predicated on broadly delivered MDA will interrupt transmission without also improving hygiene infrastructure. In fact, most of the benefits attributed to cMDA amongst these cadres was driven by beliefs that MDA will be more acceptable when delivered in the community, as opposed to its potential for interrupt transmission. Although evidence regarding the effectiveness of WASH on STH transmission interruption is still weak, it is also important to monitor how implementer and community enthusiasm for cMDA changes over time, potentially driven by the absence of WASH interventions (32).

Participants across all geographic areas noted that myths and rumors could pose serious challenges to cMDA delivery, adding further to the literature documenting this obstacle in other settings (10, 26, 33, 34). Children in Benin and Malawi noted specific concerns about side effects of deworming treatment circulating within schools prior to and post-MDA campaigns. While side effects for albendazole are typically quite mild, albendazole is often co-administered to children with praziquantel as preventative chemotherapy for schistosomiasis. Praziquantel can cause relatively more severe side effects, including diarrhea and vomiting (35). Thus, prior experiences with MDA campaigns including other treatments

For peer review only - http://bmjopen.bmj.com/site/about/guidelines.xhtml

might influence future perceptions about albendazole specifically or MDA generally (36). Evidence suggests that effective health workers can overcome these individual-level perceptions (31); addressing myths and rumors will require targeted and pro-active community sensitization and CDD training that openly discuss local myths and rumors pre-intervention.

Mistrust toward public health campaigns and government-run health programs was prevalent across settings. These concerns were driven by prior negative experiences with medical interventions and programs in which limited information was provided in advance of treatment, parents were minimally engaged in school-based MDA, and there were perceived concerns about drug quality. Potential strategies for overcoming these barriers include engaging local leaders (29, 33, 37), targeted education campaigns (26, 38, 39), community mobilization (37, 40, 41), and engagement of trusted, trained personnel to administer preventive treatment.

For peer review only

During the design of our initial question guide (Appendix 1), we drew from selected constructs across all CFIR domains. However, during data analysis no constructs within the outer setting emerged as major facilitators or barriers to launch of cMDA campaigns. The outer setting domain is comprised of constructs representing external influences on implementation and, perhaps because these data were collected at the community-level, respondents were more focused on individuals involved in implementation and implementation processes (18). Additionally, because we conducted a formative study we did not link identified implementation determinants to observed implementation outcomes, however subsequent data collection activities in DeWorm3—once the trial is underway and outcome data are collected—will afford these opportunities (20).

This study had several limitations. Some participants may have heard about DeWorm3 before participating in FGDs, which may have contributed to social desirability or response biases. While a large

For peer review only - http://bmjopen.bmj.com/site/about/guidelines.xhtml

number of FGDs were conducted across heterogeneous settings, it is also possible that the study findings may not be generalizable to other STH-endemic areas.

Conclusion

This study supports that cMDA, particularly as an alternative to school-based MDA, is generally acceptable across heterogenous settings and builds upon the existing literature exploring facilitators and barriers of launching and implementing cMDA. Community engagement including STH education, understanding preferred distribution times and methods, involvement of local leaders and familiar health workers or CDDs are critical for implementation success. Potential barriers, including mistrust of free drug distribution, fear of side effects, and limited perceived need of treatment can be addressed through community sensitization and engagement of local leaders and trusted health workers. These findings were used to shape implementation activities during the DeWorm3 trial, in order to ensure high acceptability of the intervention and high cMDA coverage from the onset of the trial. Formative research exploring attitudes and community-derived recommendations should be conducted when possible to improve community acceptability of new interventions.

Author contributions

ARM, JLW, MCGC, SSRA, KK, and MI conceived of the initial study design. EA, SPK, FC, CIT, PN, MI, KK, KA, AT, and YJ were involved in data collection and data processing. AR managed the data. AR, ABE, SL, and ARM contributed to data analysis. SL, EA, and ARM led the writing of the paper. All authors read and approved the final version of the manuscript.

Competing Interests

None declared.

Funding statement

For peer review only

The DeWorm3 study was funded by the Bill & Melinda Gates Foundation (https://www.gatesfoundation.org/) and (grant number OPP1129535). The funders had no role in study design, data collection and analysis, decision to publish, or preparation of the manuscript.

Data availability

Data are available from the DeWorm3 Institutional Data Access Committee (contact dw3data@uw.edu) for researchers who meet criteria for data access.

Acknowledgements

The authors wish to thank all of the study participants, communities and community leaders, national NTD program staff and other local, regional and national partners who have participated or supported the DeWorm3 study. We also would like to acknowledge the work of all members of the DeWorm3 study teams and affiliated institutions. We give special thanks to Rachel Geyer, Elodie Yard, and Mira Emmanuel-Fabula for contributions to data collection, management, or analysis. We thank Rajeshkumar Rajendran, Surya Kulasekaran and Chinnaduraipandi Paulsamy for their support in data collection in the field sites in India. We also thank Adolphe Kpatchavi, Roch Houngnihan and Abdoulaye Benon Monra for their support during the training of surveyors in the Benin site.

For peer review only

References

1. Hotez PJ, Molyneux DH, Fenwick A, Kumaresan J, Sachs SE, Sachs JD, et al. Control of neglected tropical diseases. New England Journal of Medicine. 2007;357(10):1018-27.
2. Jamison D, Breman J, Measham A, Alleyne G, Claeson M, Evans D, et al. Helminth Infections: Soil-transmitted Helminth Infections and Schistosomiasis--Disease Control Priorities in Developing Countries. 2006.
3. (WHO) WHO. Guideline on when to start antiretroviral therapy and on pre-exposure prophylaxis for HIV. 2015.
4. Brooker SJ, Nikolay B, Balabanova D, Pullan RL. Global feasibility assessment of interrupting the transmission of soil-transmitted helminths: a statistical modelling study. The Lancet infectious diseases. 2015;15(8):941-50.
5. Pullan RL, Halliday KE, Oswald WE, Mcharo C, Beaumont E, Kepha S, et al. Effects, equity, and cost of school-based and community-wide treatment strategies for soil-transmitted helminths in Kenya: a cluster-randomised controlled trial. The Lancet. 2019;393(10185):2039-50.
6. Lo NC, Bogoch II, Blackburn BG, Raso G, N'Goran EK, Coulibaly JT, et al. Comparison of community-wide, integrated mass drug administration strategies for schistosomiasis and soil-transmitted helminthiasis: a cost-effectiveness modelling study. The Lancet Global Health. 2015;3(10):e629-e38.
7. Farrell SH, Truscott JE, Anderson RM. The importance of patient compliance in repeated rounds of mass drug administration (MDA) for the elimination of intestinal helminth transmission. Parasites & vectors. 2017;10(1):291.
8. Shuford KV, Turner HC, Anderson RM. Compliance with anthelmintic treatment in the neglected tropical diseases control programmes: a systematic review. Parasites & vectors. 2016;9(1):29.
9. Yajima A, Mikhailov A, Mbabazi PS, Gabrielli AF, Minchiotti S, Montresor A, et al. Preventive Chemotherapy and Transmission Control (PCT) databank: a tool for planning, implementation and monitoring of integrated preventive chemotherapy for control of neglected tropical diseases. Transactions of the Royal Society of Tropical Medicine and Hygiene. 2012;106(4):215-22.
10. Krentel A, Fischer PU, Weil GJ. A review of factors that influence individual compliance with mass drug administration for elimination of lymphatic filariasis. PLoS Negl Trop Dis. 2013;7(11):e2447.
11. Gyapong JO, Owusu IO, Vroom FBd-C, Mensah EO, Gyapong M. Elimination of lymphatic filariasis: current perspectives on mass drug administration. Research and reports in tropical medicine. 2018;9:25.
12. Silumbwe A, Halwindi H, Zulu JM. How community engagement strategies shape participation in mass drug administration programmes for lymphatic filariasis: The case of Luangwa District, Zambia. PLoS Negl Trop Dis. 2019;13(11):e0007861.
13. Stetler C, Legro M, Wallace C, Bowman C, Guihan M, Hagedorn H, et al. The role of formative evaluation in implementation research and the QUERI experience. Journal of general internal medicine and the QUERI experience. Journal of General Internal Medicine. 2006;21(2):S1-8.
14. Lugtenberg M, Burgers J, Westert G. Effects of evidence-based clinical practice guidelines on quality of care: a systematic review. BMJ Quality & Safety. 2009;18(5):385-92.
15. Ásbjörnsdóttir KH, Ajjampur SSR, Anderson RM, Bailey R, Gardiner I, Halliday KE, et al. Assessing the feasibility of interrupting the transmission of soil-transmitted helminths through mass drug administration: The DeWorm3 cluster randomized trial protocol. PLoS neglected tropical diseases. 2018;12(1).

For peer review only - http://bmjopen.bmj.com/site/about/guidelines.xhtml

16. Curran GM, Bauer M, Mittman B, Pyne JM, Stetler C. Effectiveness-implementation hybrid designs: combining elements of clinical effectiveness and implementation research to enhance public health impact. Medical care. 2012;50(3):217.
17. Means AR, Ajjampur SS, Bailey R, Galactionova K, Gwayi-Chore M-C, Halliday K, et al. Evaluating the sustainability, scalability, and replicability of an STH transmission interruption intervention: The DeWorm3 implementation science protocol. PLoS neglected tropical diseases. 2018;12(1):e0005988.
18. Damschroder LJ, Aron DC, Keith RE, Kirsh SR, Alexander JA, Lowery JC. Fostering implementation of health services research findings into practice: a consolidated framework for advancing implementation science. Implement Sci. 2009;4:50.
19. Means A, Kemp C, Gwayi-Chore M, Gimbel S, Soi C, Sherr K, et al. Evaluating and optimizing the consolidated framework for implementation research (CFIR) for use in low-and middle-income countries: a systematic review. Implementation Science. 2020;15(1):1-9.
20. Kirk MA, Kelley C, Yankey N, Birken SA, Abadie B, Damschroder L. A systematic review of the use of the consolidated framework for implementation research. Implementation Science. 2015;11(1):72.
21. Cassell C, Symon G. Essential guide to qualitative methods in organizational research: Sage; 2004.
22. Nowell LS, Norris JM, White DE, Moules NJ. Thematic analysis: Striving to meet the trustworthiness criteria. International Journal of Qualitative Methods. 2017;16(1):1609406917733847.
23. Babu BV, Satyanarayana K. Factors responsible for coverage and compliance in mass drug administration during the programme to eliminate lymphatic filariasis in the East Godavari District, South India. Trop Doct. 2003;33(2):79-82.
24. Gunawardena S, Ismail M, Bradley M, Karunaweera N. Factors influencing drug compliance in the mass drug administration programme against filariasis in the Western province of Sri Lanka. Transactions of the Royal Society of Tropical Medicine and Hygiene. 2007;101(5):445-53.
25. Ahorlu CSK, Koka E, Adu-Amankwah S, Otchere J, de Souza DK. Community perspectives on persistent transmission of lymphatic filariasis in three hotspot districts in Ghana after 15 rounds of mass drug administration: a qualitative assessment. BMC public health. 2018;18(1):238.
26. Legge H, Kepha S, Prochazka M, Halliday K, Pullan R, Gwayi-Chore MC, et al. Implementer and recipient perspectives of community-wide mass drug administration for soil-transmitted helminths in Kwale County, Kenya. PLoS Negl Trop Dis. 2020;14(4):e0008258.
27. Babu BV, Mishra S. Mass drug administration under the programme to eliminate lymphatic filariasis in Orissa, India: a mixed-methods study to identify factors associated with compliance and non-compliance. Transactions of the Royal Society of Tropical Medicine and Hygiene. 2008;102(12):1207-13.
28. Dial NJ, Ceesay SJ, Gosling RD, D'Alessandro U, Baltzell KA. A qualitative study to assess community barriers to malaria mass drug administration trials in The Gambia. Malaria Journal. 2014;13(1):47.
29. Wanzira H, Naiga S, Mulebeke R, Bukenya F, Nabukenya M, Omoding O, et al. Community facilitators and barriers to a successful implementation of mass drug administration and indoor residual spraying for malaria prevention in Uganda: a qualitative study. Malar J. 2018;17(1):474.
30. Lahariya C, Mishra A. Strengthening of mass drug administration implementation is required to eliminate lymphatic filariasis from India: an evaluation study. J Vector Borne Dis. 2008;45(4):313-20.
31. Kisoka WJ, Simonsen PE, Malecela MN, Tersbøl BP, Mushi DL, Meyrowitsch DW. Factors influencing drug uptake during mass drug administration for control of lymphatic filariasis in rural and urban Tanzania. PLoS One. 2014;9(10):e109316.
32. Nery SV, Pickering AJ, Abate E, Asmare A, Barrett L, Benjamin-Chung J, et al. The role of water, sanitation and hygiene interventions in reducing soil-transmitted helminths: interpreting the evidence and identifying next steps. Parasites & vectors. 2019;12(1):273.
33. Gyapong JO, Owusu IO, da-Costa Vroom FB, Mensah EO, Gyapong M. Elimination of lymphatic filariasis: current perspectives on mass drug administration. Res Rep Trop Med. 2018;9:25-33.

For peer review only - http://bmjopen.bmj.com/site/about/guidelines.xhtml

34. Kisoka WJ, Tersbol BP, Meyrowitsch DW, Simonsen PE, Mushi DL. Community Members' Perceptions of Mass Drug Administration for Control of Lymphatic Filariasis in Rural and Urban Tanzania. J Biosoc Sci. 2016;48(1):94-112.
35. Sousa-Figueiredo JC, Pleasant J, Day M, Betson M, Rollinson D, Montresor A, et al. Treatment of intestinal schistosomiasis in Ugandan preschool children: best diagnosis, treatment efficacy and side-effects, and an extended praziquantel dosing pole. International Health. 2010;2(2):103-13.
36. Musuva RM, Matey E, Masaku J, Odhiambo G, Mwende F, Thuita I, et al. Lessons from implementing mass drug administration for soil transmitted helminths among pre-school aged children during school based deworming program at the Kenyan coast. BMC public health. 2017;17(1):575.
37. Omedo MO, Matey EJ, Awiti A, Ogutu M, Alaii J, Karanja DM, et al. Community health workers' experiences and perspectives on mass drug administration for schistosomiasis control in western Kenya: the SCORE Project. The American journal of tropical medicine and hygiene. 2012;87(6):1065-72.
38. Cantey PT, Rout J, Rao G, Williamson J, Fox LM. Increasing compliance with mass drug administration programs for lymphatic filariasis in India through education and lymphedema management programs. PLoS Negl Trop Dis. 2010;4(6):e728.
39. Garfield RM, Vermund SH. Health education and community participation in mass drug administration for malaria in Nicaragua. Social Science & Medicine. 1986;22(8):869-77.
40. De Martin S, Von Seidlein L, Deen JL, Pinder M, Walraven G, Greenwood B. Community perceptions of a mass administration of an antimalarial drug combination in The Gambia. Tropical Medicine & International Health. 2001;6(6):442-8.
41. Mulugeta A, Gebregergs GB, Asfaw S, Yemane D, Mitiku M, Meresa B, et al. Coverage, social mobilization and challenges of mass Zithromax administration campaign in South and South East zones of Tigray, Northern Ethiopia: A cross sectional study. PLoS neglected tropical diseases. 2018;12(2):e0006288.

For peer review only

For peer review only - http://bmjopen.bmj.com/site/about/guidelines.xhtml

Appendix 1. Qualitative interview guides

Interview guide #1: Health Centre & DeWorm3 Supervisory Teams

	Question
**1**	What kind of information or evidence are you aware of that shows whether or not community-wide MDA for interrupting transmission of STH will work in your setting?<br><br>*Follow-up questions:*<br>• *Is this evidence strong or weak?*<br>• *What evidence are you aware of from your own research? Practice guidelines? Published literature? Other settings?*<br>• *How does this knowledge affect your perception of the intervention?*
**2**	What advantages does community-wide MDA for STH have compared to school-age targeted programs? What disadvantages?
**3**	When delivering community-wide MDA for STH, what local adaptations should be made so that the intervention is effective?<br><br>*Follow-up questions:*<br>• *What aspects of the intervention should not be adapted?*<br>• *Do you think you will be able to make these adaptations? Why or why not?*
**4**	How complicated is delivery of the entire community-wide MDA for STH intervention? Please consider the following aspects of the intervention: duration of an MDA round, target population, and number of steps and sub-activities involved to fully implement.
**5**	What supportive materials or tools are needed to ensure effective implementation of community-wide MDA for STH?
**6**	What community-wide MDA for STH activities are built upon the previous LF MDA program infrastructure and experiences?<br><br>*Follow-up questions:*<br>• If none, what LF program activities could be leveraged for community-wide MDA for STH?
**7**	From your perspective, what are unique costs of implementing community-wide MDA for STH, relative to the standard of care of school-age targeted MDA?
**8**	What barriers might community members face in participating in community-wide MDA for STH?<br><br>*Follow-up question:*<br>• *What could be done to overcome these barriers?*
**9**	What kinds of infrastructure changes to the health system will be needed to accommodate community-wide MDA for STH?<br><br>*Follow-up questions:*

	Question
	• *If community-wide MDA for STH is scaled-up after the DeWorm3 project, will there need to be any changes in formal policies? Changes in information systems or data reporting systems? Other?*<br>• *What kind of approvals do you think will be needed to transition the STH program entirely to community-wide MDA? Who will need to be involved?*<br>• *Can you describe the process that will be needed to make these changes?*
**10**	How accepting are your co-workers of implementing community-wide MDA for STH? Why?
**11**	Do you think community-wide MDA for STH could be integrated into routine MOH programs?<br><br>*Follow-up questions:*<br>• If yes, how? If no, why not?<br>• *What would the effects of integration on routine programs be?*
**12**	How important do you think it is to implement community-wide MDA for STH compared to the other health priorities?
**13**	Do you feel incentivized to ensure that the DeWorm3 trial is successful in interrupting transmission of STH? What is the incentive?
**14**	How supportive are government and non-governmental leadership of implementing community-wide MDA for STH?<br><br>*Follow-up question:*<br>• *How do you think it will affect uptake/implementation?*
**15**	What kind of relevant training will you have during this intervention? Do you feel the training will prepare you to carry out the roles and responsibilities expected of you? How so?<br><br>*Follow-up questions:*<br>• *What are the positive aspects of the training?*<br>• *What is missing?*<br>• *What kind of continued training is necessary?*
**16**	Do you think that community-wide MDA could interrupt STH transmission (i.e. stop the spread of intestinal worms) in your setting? Why or why not?
**17**	How confident are you that you will be able to successfully carry out your DeWorm3 related duties? What gives you that level of confidence (or lack of confidence)?
**18**	Can you describe your team's plan for implementing community-wide MDA for STH?<br><br>*Follow-up questions:*<br>• *Do you think everyone involved understands the plan well, or is it too complicated?*<br>• *What do you do if you have to modify or revise the plan due to challenges, errors, or mistakes?*
**19**	Who are the key influential individuals or organizations to get buy-in from during the DeWorm3 study?

For peer review only

	Question
**20**	How should community members be informed about community-wide MDA or other DeWorm3 interventions before they occur?
**21**	What are some strategies to achieve high MDA treatment coverage?
**22**	Is there any other information that you would like to share about community-wide MDA today?

Interview Guide #2: Community Drug Distributors & Community Health Workers

#	Question
**1**	What advantages does community-wide MDA for STH have compared to school-age targeted programs? What disadvantages?
**2**	When delivering community-wide MDA for STH, what local adaptations should be made so that the intervention is effective?<br>*Follow-up questions:*<br>• What aspects of the intervention should not be adapted?<br>• Do you think you will be able to make these adaptations? Why or why not?
**3**	How complicated is delivery of the entire community-wide MDA for STH intervention? Please consider the following aspects of the intervention: duration of an MDA round, target population, and number of steps and sub-activities involved to fully implement.
**4**	What supportive materials or tools are needed to ensure effective implementation of community-wide MDA for STH?
**5**	From your perspective, what are unique costs of implementing community-wide MDA for STH, relative to the standard of care of school-age targeted MDA?
**6**	What barriers might community members face in participating in community-wide MDA for STH?<br>*Follow-up question:*<br>• *What could be done to overcome these barriers?*
**10**	How accepting are your co-workers of implementing community-wide MDA for STH? Why?
**11**	Do you think community-wide MDA for STH could be integrated into routine MOH programs?<br>*Follow-up questions:*<br>• *If yes, how? If no, why not?*<br>• *What would the effects of integration on routine programs be?*
**12**	How important do you think it is to implement community-wide MDA for STH compared to the other health priorities?
**13**	Do you feel incentivized to ensure that the DeWorm3 trial is successful in interrupting transmission of STH? What is the incentive?
**14**	How supportive are government and non-governmental leadership of implementing community-wide MDA for STH?

#	Question
	Follow-up question:<br>• *How do you think it will affect uptake/implementation?*
**15**	What kind of relevant training will you have during this intervention? Do you feel the training will prepare you to carry out the roles and responsibilities expected of you? How so?<br><br>*Follow-up questions:*<br>• *What are the positive aspects of the training?*<br>• *What is missing?*<br>• *What kind of continued training is necessary?*
**16**	Do you think that community-wide MDA could interrupt STH transmission (i.e. stop the spread of intestinal worms) in your setting? Why or why not?
**17**	How confident are you that you will be able to successfully carry out your DeWorm3 related duties? What gives you that level of confidence (or lack of confidence)?
**18**	Can you describe your team's plan for implementing community-wide MDA for STH?<br><br>*Follow-up questions:*<br>• *Do you think everyone involved understands the plan well, or is it too complicated?*<br>• *What do you do if you have to modify or revise the plan due to challenges, errors, or mistakes?*
**19**	Who are the key influential individuals or organizations to get buy-in from during the DeWorm3 study?
**20**	How should community members be informed about community-wide MDA or other DeWorm3 interventions before they occur?
**21**	What are some strategies to achieve high MDA treatment coverage?
**22**	Is there any other information that you would like to share about community-wide MDA today?

Interview Guide #3: Community members

#	Question
**1**	What advantages does community-wide MDA for STH have compared to school-age targeted programs?<br><br>*Follow-up question:*<br>• *What disadvantages?*
**2**	When delivering community-wide MDA for STH, what local adaptations should be made so that the intervention is effective?<br><br>*Follow-up question:*<br>• *What aspects of the intervention should not be adapted?*

#	Question
**3**	What barriers might community members face in participating in community-wide MDA for STH?<br><br>*Follow-up question:*<br>• *What could be done to overcome these barriers?*
**4**	Do you remember the lymphatic filariasis (LF) MDA programs that used to occur in this area? What do you remember about them?<br><br>*Follow-up questions:*<br>• *What could be done to overcome these barriers?*<br>• *Did you participate in those treatment days by swallowing the medicines given to you? Why or why not?*<br>• *Has that affected your interest in participating in the current mass treatment days?*
**5**	Do you think that community-wide MDA could interrupt STH transmission (i.e. stop the spread of intestinal worms) in your setting? Why or why not?
**6**	Who are the key influential individuals or organizations to get buy-in from during the DeWorm3 study?<br><br>*Follow-up questions:*<br>• *Can you provide specific examples of why you think their buy-in is important?*
**7**	How should community members be informed about community-wide MDA or other DeWorm3 interventions before they occur?
**8**	What are some strategies to achieve high MDA treatment coverage?
**9**	Is there any other information that you would like to share about community-wide MDA today?

review only
